# Urbanisation and Lockdown Impact on Airborne Fungal Communities in Tropical Landscapes: A Comparative Study of Urban and Peri‐Urban Environments

**DOI:** 10.1111/1758-2229.70078

**Published:** 2025-05-13

**Authors:** Euler Gallego‐Cartagena, Wendy Morgado‐Gamero, Iuleder de Moya‐Hernández, Carlos Díaz‐Uribe, Alexander Parody, Héctor Morillas, Brayan Bayona‐Pacheco, Gabrielle Pellegrin, Dayana Agudelo‐Castañeda

**Affiliations:** ^1^ Department of Civil and Environmental Universidad de la Costa Barranquilla Colombia; ^2^ Department of Exact and Natural Sciences Universidad de la Costa Barranquilla Colombia; ^3^ Department of Biology McGill University Montreal Quebec Canada; ^4^ Chemistry Program, Faculty of Basic Sciences Universidad del Atlántico Puerto Colombia Colombia; ^5^ Faculty of Engineering Universidad Libre Barranquilla Colombia; ^6^ Department of Didactic of Mathematics, Experimental and Social Sciences, Faculty of Education and Sport University of the Basque Country Vitoria‐Gasteiz Spain; ^7^ Department of Medicine, Division of Health Sciences Universidad del Norte Barranquilla Colombia; ^8^ IRD Institut de recherche pour le développement Montpellier France; ^9^ Department of Civil and Environmental Engineering Universidad del Norte Barranquilla Colombia

**Keywords:** Bayesian neural network, environmental monitoring, fungal bioaerosols, respiratory health risks, spatiotemporal distribution, tropical urban environments

## Abstract

This study assessed the concentration, composition, and spatiotemporal distribution of airborne fungi in a metropolitan area, comparing urban and peri‐urban sites across rainy and dry seasons. An 8‐month fungal bioaerosol monitoring was conducted using a six‐stage Andersen cascade impactor. Data analysis involved generalised linear regression models and multifactorial ANOVA to assess the relationships between meteorological conditions, sampling sites, campaigns, fungal concentrations, and impactor stages. Additionally, a Bayesian neural network was developed to predict bioaerosol dynamics based on the analysed variables. We identified 10 viable fungal species, including *Aspergillus niger*, *Aspergillus nidulans*, *Aspergillus* *. fumigatus* , *Aspergillus terreus*, *Aspergillus flavus*, *Aspergillus versicolor*, *Penicillium* spp. and *Fusarium oxysporum*. Notable differences in the aerodynamic sizes of fungal particles influenced their distribution and potential impact on the respiratory system. The Bayesian neural network successfully predicted fungal bioaerosol concentrations with an accuracy of 76.87%. Our findings reveal the significant role of environmental and human‐related factors in shaping bioaerosol distribution in tropical urban contexts. This research provides essential insights into the behaviour of fungal bioaerosols, highlighting their relevance for public health, especially for immunocompromised populations, and their impact on local agriculture. Furthermore, it demonstrates the potential of fungal bioaerosols as bioindicators for environmental monitoring and predictive modelling.

## Graphical abstract

This study assessed the distribution of fungal bioaerosols in tropical urban and peri‐urban environments. Using a six‐stage Andersen cascade impactor and a Bayesian neural network, fungal concentrations were predicted with an accuracy of 76.87%. It highlights the role of environmental factorsin fungal dispersion, associated respiratory risks, and bioaerosols as environmental indicators, emphasising their importance for public health and regulating atmospheric biocontamination in these regions.

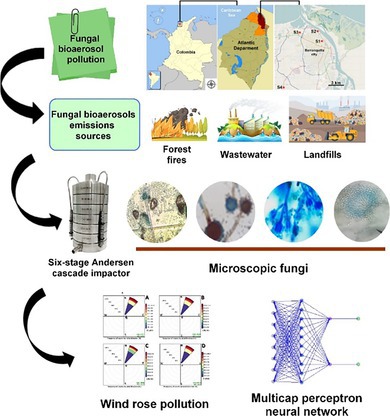

## 1 Introduction

Air pollution has become a growing concern for the scientific community due to its negative impact on human health. The World Health Organisation (WHO) has indicated that most cities worldwide experience high air pollution levels, which have been associated with direct and indirect health damage (Maji and Namdeo 2021; Metelmann et al. 2021). These elevated pollution levels in various regions, especially in large cities in Latin America, Asia, and Africa, have been linked to deaths from respiratory and cardiovascular diseases (Gómez‐Dantés et al. 2016; Awokola et al. 2020; Gouveia et al. 2018; Amarillo and Carreras 2012; Herrera et al. 2016; Herrera and Cabrera‐Barona 2022; Rodriguez‐Villamizar et al. 2022; Rodríguez‐Villamizar et al. 2021; Agudelo‐Castañeda et al. 2019). Moreover, according to the WHO, outdoor air pollution causes approximately 2.9 million premature deaths (Cohen et al. 2017; Bui et al. 2023).

Another significant health risk factor related to air pollution is the presence of bioaerosols, also known as primary biological aerosol particles (PBAP) (Després et al. 2012). PBAP includes viable and non‐viable particles derived from living organisms, such as bacteria, fungal spores, pollen, mites, and dead tissues (Emygdio et al. 2018). In contrast, fungal spores dominate the total bioaerosol composition (Kakde 2012). The release of PBAP occurs mainly during processes involving biological materials, generating sufficient energy to release small particles from a more considerable substance, such as wind, water, or mechanical movement (Fung and Hughson 2003; Kakde 2012). Furthermore, it is widely recognised that climatic conditions, such as temperature, rainfall, humidity, and air currents, profoundly influence the development, propagation, and survival of bioaerosols (Lee et al. 2010; Gong et al. 2020; Jeong et al. 2022), contributing to approximately a quarter of aero‐transported biogenic materials (Šantl‐Temkiv et al. 2020). PBAP is an inseparable part of human society and can be found in various environments where bacteria and fungi are predominant (D'Arcy et al. 2014).

Numerous studies have demonstrated the harmful impact of bioaerosols on human health, causing toxic, allergic, or irritative disorders depending on characteristics such as size, microbial gender, or species (Fung and Hughson 2003; Laumbach et al., Laumbach and Kipen 2005; Kim et al. 2018; Robichaud 2020). Therefore, it is crucial to characterise microbial dispersion and assess the potential risk of microbial aerosols in outdoor environments (Madsen et al. 2021). In the last decade, a significant amount of research has been conducted to quantify the concentrations and size distributions of bioaerosols in different environmental scenarios, such as bird breeding sites (Nguyen et al. 2021), landfills (Li et al. 2021; Madhwal et al. 2020a; Madhwal et al. 2020b; Morgado‐Gamero et al. 2021), clinics or hospitals (Liu et al. 2021; Pastuszka et al. 2005), and wastewater treatment plants (Bruni et al. 2020; Korzeniewska 2011). In particular, fungal bioaerosols can potentially cause allergies, infections, and intoxications (Morgado Gamero, Agudelo‐Castañeda, et al. 2018; Morgado Gamero, Ramírez, et al. 2018).

Due to the spore's fungus, which can readily travel through the air as bioaerosols and deposit in any tissue, infections in humans and plants can be seen. In the same way, the influence of global warming, natural disasters, the use of fungicides, and exposure to chemicals that cause stress to fungi is forcing them to change, become resistant, and achieve adaptation that facilitates their spread in environments where they were not before (Seidel et al. 2024; Verweij et al. 2022; Konkel Neabore 2024).

In indoor environments such as office buildings, hospitals, dormitories, airplanes, and air‐conditioned structures that heavily rely on air recirculation, abnormally high quantities of viable bioaerosols can be found, often including pathogenic bioaerosols (Norouzian Baghani et al. 2021; Jeong et al. 2022; Li et al. 2015). On the other hand, outdoor mould tends to proliferate in various environments, although its concentration and composition in the air vary with the season. Additionally, spore counts may fluctuate throughout the day, typically higher in the afternoon and early evening (Green et al. 2006).

According to the Global Action For Fungal Infections (GAFFI), approximately 6.55 million patients develop potentially fatal fungal infections annually, of which, unfortunately, 3.75 million patients die, associated with some immunosuppressive disease. It is essential to highlight the respiratory and invasive impact of fungi, especially mentioning the genus *Aspergillus*; for example, the group GAFFI reports that there is an annual incidence of 1.837.000 affected in chronic pulmonary aspergillosis, 2,100,000 in invasive aspergillosis, and 11,500,000 in fungal asthma (Bongomin et al. 2017; Denham et al. 2019).

Filamentous fungi, such as *Penicillium* spp., *Aspergillus* spp., *Mucor* spp. and *Rhizopus* spp., are commonly associated with allergies, infections, irritations, and toxicity (Stetzenbach et al. 2004; Schwab and Straus 2004; Fung and Hughson 2003; Mentese et al. 2009). Among these fungi, *Aspergillus* spp., *Cladosporium* spp. and *Penicillium* spp. are the most frequently associated with allergies, and they exist in both indoor and outdoor environments (Esch et al. 2020; Fung and Hughson 2003; Wei et al. 1993). The threshold concentrations required to trigger allergic reactions to these species are unknown, but increased airborne concentrations have been associated with a higher risk of respiratory arrest in sensitised patients. Fungal bioaerosol high exposure may also coincide with the season of peak grass and weed pollen, affecting fungal‐allergic patients who, in many cases, are also sensitised to other aeroallergens (Singh and Hays 2016). It is noteworthy to mention the urgent need to evaluate the prevalence of different species of filamentous fungi, both in human exposure and at the agricultural level. For example, the genus *Aspergillus* is characterised by its wide distribution in the environment due to its high sporulation capacity, which raises concerns about its ability to infect susceptible hosts, such as individuals with haematological malignancies (Raposo Puglia et al. 2024), cancer (Wan et al. 2024), diabetes mellitus (Li et al. 2024), lung transplant recipients (Walti et al. 2024), lung cancer (Whittaker et al. 2024), HIV‐positive individuals (Denning and Morgan 2022), premature newborns (Mohammad et al. 2024), and leukaemia patients (Penack et al. 2024). Similarly, *Fusarium oxysporum* is a filamentous, saprophytic fungus that can grow and survive for long periods in organic matter in the soil and is characterised by being pathogenic to many plant species (Zuriegat et al. 2021). Until 2018, no case of tropical strain 4 (TR4) of *Fusarium* had been reported in Latin America. However, in 2019, the first TR4 case was reported in Colombia. In April 2021, the discovery of a banana plantation infected by Foc TR4 was confirmed in Peru, prompting all Latin American countries to constantly monitor their crops (García‐Bastidas et al. 2020; Olivares et al. 2021).

The assessment of airborne fungi, both indoors and outdoors, is crucial in establishing criteria and indicators of atmospheric biocontamination, which is essential in designing technologies and methods related to the prevention and mitigation of bioaerosol release and exposure. Additionally, knowledge of atmospheric biocontamination is vital for decision‐makers in environmental health since outdoor air quality influences indoor air quality through the penetration of air from outdoors to indoors, mainly depending on ventilation efficiency (Lee and Yoo 2022; Mentese et al. 2009). The contribution of outdoor air to indoor air pollution depends on the concentration and composition of bioaerosols in the outdoor air.

Studies on airborne fungi levels in Colombia in outdoor settings are scarce (Huertas et al. 2018; Morgado Gamero, Agudelo‐Castañeda, et al. 2018; Morgado Gamero, Ramírez, et al. 2018; Morgado‐Gamero et al. 2021). Cities like Bogotá and Medellín exhibit high levels of air pollution, with Bogotá being the most polluted city, exceeding WHO guidelines for PM_10_ and PM_2.5_ (Hernández‐Flórez et al. 2013; Rodríguez‐Camargo et al. 2020). In contrast, Barranquilla has experienced rapid urban, population, and industrial growth over the past decade. It is a focal point for preventing and mitigating air quality deterioration in tropical urban environments (Gallego‐Cartagena et al. 2022). However, official reports from Colombia's National Department of Statistics and the Integrated System of Social Protection Information indicate that the primary cause of mortality in Barranquilla is related to acute respiratory infections (Betancur‐Otalvaro et al. 2023; Amaya Díaz et al. 2021).

Despite the implementation of regulatory instruments and monitoring/control systems to reduce air pollution, difficulties persist in establishing standardised methodologies for monitoring and controlling the levels of atmospheric biocontamination. Hence, it is essential to recognise the activities or processes related to the dynamics and sources of bioaerosols in open urban environments to design technologies and methods that contribute to the prevention and mitigation of PBAP release and exposure.

This study assessed the concentration, composition, and spatiotemporal distribution of airborne fungi in a metropolitan area during the rainy and dry seasons. A six‐stage Andersen Cascade Impactor was used to collect airborne fungi in both morning and afternoon sessions, enabling us to determine the size of viable particles in the human respiratory system. Additionally, the study provided valuable insights into the behaviour of fungal bioaerosols in urban and rural environments within a tropical city. Furthermore, implementing a Bayesian neural network was crucial in establishing a solid foundation for our predictive capabilities based on current bioaerosol monitoring techniques, focusing on exploring the potential of fungal bioaerosols as environmental bioindicators.

## 2 Materials and Methods

### 2.1 Study Area, Climatic Conditions, and Sampling Sites

**FIGURE 1:**
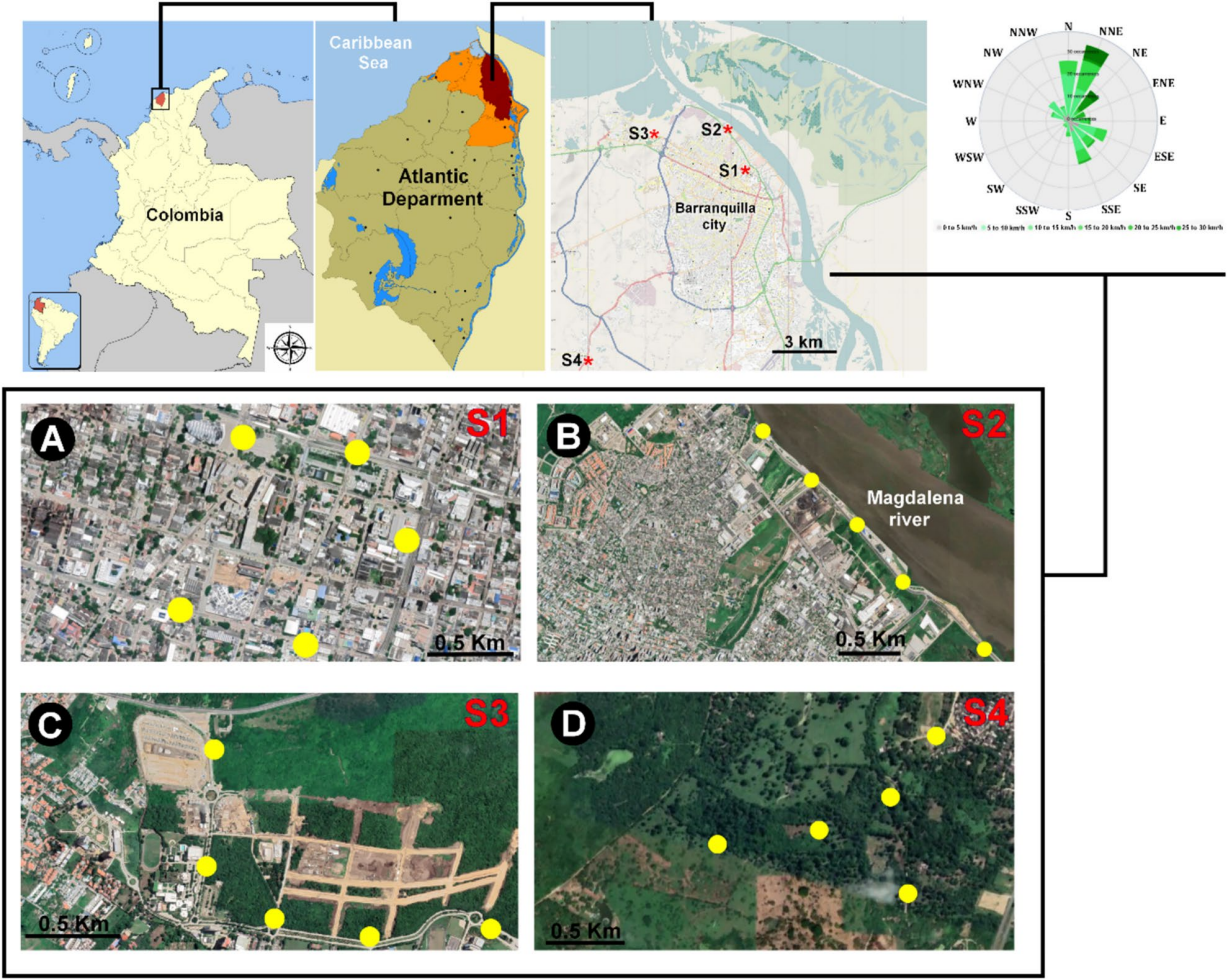
Location of sampling sites in Barranquilla City, Colombia: (A) Peace Square (S1); (B) Barranquilla Riverwalk (S2); (C) Puerto Colombia (S3); (D) Galapa (S4).

Airborne fungi were sampled in the urban area of Barranquilla, the capital of the Atlántico Department in Colombia (Figure 1), located on the western bank of the Magdalena River, 7.5 km from its mouth into the Caribbean Sea, covering an area of 154 km^2^ with a population of 2,239,103 inhabitants (DANE 2021). This urban center hosts pharmaceutical, chemical, food, construction materials, metallurgical, bodywork, and shipyard industries. Barranquilla has a predominantly tropical savanna climate according to the Köppen‐Geiger climate classification, with an average annual temperature of 27.1°C. The minimum temperatures occur in February, reaching 25.2°C, and the maximum in July, reaching 29.4°C. Moderate wind flows prevail from the northeast (42.7%) and north (25%), with occasional observations from the east, southeast, and south, accounting for 5.8%, 6.1%, and 6.1% of the total observations, respectively (IDEAM 2023). The average annual rainfall is 814 mm, with the highest peak in October (1396 mm) and the lowest in February (22 mm). The annual relative humidity (% RH) was approximately 80%, reaching its highest peak in October (85.10%) and lowest in February (78.21%). The mean annual solar luminosity is approximately 3406.8 h (Agudelo‐Castañeda et al. 2020; IDEAM 2023).

The sampling sites were selected based on a combination of environmental factors (e.g., wind direction, humidity conditions and predominant flora) and anthropogenic factors (e.g., industrial activities, vehicular traffic, stream channels and wastewater discharges) that potentially contribute to the generation, dynamics, and distribution of airborne fungi (Gallego‐Cartagena et al. 2020).

Site S1, *Peace Square*, is located in the city's Historic Center (10°59′16″ N, 74°47′20″ W). This site is 50 m from a high‐traffic road, 100 m from an urban bus station, and 500 m from the city's industrial complex. Additionally, S1 serves as a gathering point for public demonstrations and cultural events and is surrounded by residential buildings, wastewater channels, and recreational areas (Figure 1A).

Site S2, *Barranquilla Riverwalk*, is situated in the Riomar locality, along the banks of the Magdalena River, in the northeastern part of the city (10°49′0.9″ N, 74°46′13.9″ W). This relatively new area in the city is influenced by tourist activities, vehicular traffic, and its proximity to the riverbed, which is affected by the discharge of wastewater effluents 1000 m upstream or from municipalities further upstream. This contributes to this city's significant microbiological load, rich in fungi and bacteria (Figure 1B).

Site S3, the university corridor of the Sabanilla Montecarmelo district, is located in the municipality of *Puerto Colombia*, in the northwest of the metropolitan area of Barranquilla (11°01′0.05″ N, 74°51′04″ W) (Figure 1C). This site is located 1.2 km from the Ciénaga de Mallorquín, a coastal estuarine lagoon severely affected by wastewater pollution and human settlements (Fuentes‐Gándara et al. 2021; Portz et al. 2020). S3 is influenced by a wide variety of estuarine flora, an intervened tropical dry forest, and marine aerosols from the Caribbean Sea and the mouth of the Magdalena River. It should be noted that sampling points S2 and S3 are subject to the diffuse impact of recurrent forest fires that typically occur in the Vía Isla de Salamanca Park during the dry season (Nolte 2016).

To compare the fungal bioaerosol composition with the previous sampling sites, the municipality of Galapa (S4), located southwest of the metropolitan area of Barranquilla, was selected. With minimal anthropogenic influence, this site is 200 m from a natural reserve of tropical dry forest, which harbours significant ecological diversity. Thus, it is a valuable source of information on fungal bioaerosols from relatively undisturbed tropical natural systems located in a non‐urbanised area (see Figure 1D).

### 2.2 Sampling

A comprehensive monitoring strategy was designed regarding time and sampling frequency, utilising a six‐stage Andersen Cascade Impactor monitoring device and taxonomically characterising airborne fungi from different environmental scenarios. To assess the distribution of fungal aerosols between the rainy and dry seasons in the study area, samplings were conducted from October 2019 to August 2020, covering 8 months (4 months of the dry season and 4 months of the rainy season). During this period, samplings were carried out once a month, both in the morning (8:00–11:30 AM) and in the afternoon (1:30–5:00 PM), at each site. Five sampling points were selected at each location, separated by a distance of 500 m, and six samples were collected in triplicate for each site (Figure 1). The samples were collected for approximately 5 min at a rate of 28.3 L/min. In total, 90 samples were collected monthly at each site, for 2880 samples during the 8 months.

### 2.3 Instrumentation

For the sampling, a six‐stage Andersen cascade impactor (Thermo Fisher Scientific, Waltham, Massachusetts, USA) with six glass petri dishes was used (93 mm diameter). The particle sizes were fractionated into six size ranges: > 7.0 μm (stage 1), 4.7–7.0 μm (stage 2), 3.3–4.7 μm (stage 3), 2.1–3.3 μm (stage 4), 1.1–2.1 μm (stage 5) and 0.65–1.1 μm (stage 6). The sampler was mounted 1.5 m above the ground surface, which coincides with the average human inhalation zone (Jeong et al. 2022; Le and Tsai 2021). The sampling time for each sample was determined by conducting a pre‐sampling at three different time intervals (5, 10 and 15 min). After applying statistical analysis for confidence limits and concentration precision, it was established that a 5‐min sampling time showed the best precision and accuracy of concentrations at different stages of the impactor (Morgado Gamero, Agudelo‐Castañeda, et al. 2018; Morgado Gamero, Ramírez, et al. 2018).

Each sampling was conducted on a typical sunny or rainy day with similar meteorological conditions to minimise measurement uncertainty resulting from weather variations. Meteorological data were simultaneously recorded using a Kestrel Model 4500 anemometer (Boothwyn, Pennsylvania, USA) during the sample collection, which provided information on temperature, relative humidity, wind direction, and speed. During sampling, the outdoor temperature ranged from 26.2°C to 32.5°C, and the relative humidity ranged from 71.2% to 90%. The prevailing wind direction was northwest, with an average wind speed of 2.2 m/s.

### 2.4 Microbiological Analysis

Fungal aerosols were captured on Petri dishes with Sabouraud Agar Glucose culture media (Merck, Germany), described previously by (Morgado‐Gamero et al. 2019). After sampling, the agar plates were immediately transported to the laboratory and incubated at 25°C for 5 days. The sampler was disinfected with 75% ethanol to prevent contamination before and after sampling. Counting the colony‐forming units (CFU) in Petri dishes was based on their macroscopic characteristics (e.g., shape, colour, texture and border). Taxon identification was performed by staining with Lactophenol Cotton Blue (LPCB) and microscopic observation of reproductive structures and mycelium. LPCB staining is a commonly used method for identifying fungi, involving the staining of fungal structures such as spores and hyphae to enhance their visibility under the microscope (Sykes and Rankin 2014). The LPCB stain contains phenol, which kills living organisms; lactic acid, which preserves fungal structures; and cotton blue, which stains the chitin in the fungal cell walls. By observing the stained reproductive structures and mycelium under the microscope, different fungal taxa were identified based on their unique morphological characteristics. This technique enabled precise and detailed identification of fungal communities in the bioaerosols. Colony‐forming units (CFUs) on the Petri dishes were counted based on their macroscopic characteristics (e.g., shape, colour, texture and margin). The concentration of bioaerosols at each stage was determined by dividing the CFU number by the sampled volume, applying the equation defined by Thermo Fisher Scientific to the Six‐Stage Viable Andersen Cascade Impactor; the result is expressed in CFU/m^3^ (Morgado‐Gamero et al. 2021).

### 2.5 Data Treatment

The data was systematised in a spreadsheet by sampling campaigns, journeys, sampling sites, and replicates as independent variables. Data treatment was performed using the Statgraphics Centurion XVI software, using a generalised linear regression model to determine if there is a relationship between the measured meteorological variables (temperature, relative humidity, wind speed and direction) and the concentrations obtained from fungal bioaerosols with 95% confidence (*p* < 0.05). In addition, a statistical analysis was performed using a multifactorial ANOVA that established if there are significant differences between independent factors or variables, in this case, the sampling sites, the journeys, the sampling campaigns, and the stage of the Andersen impactor. A Bayesian neural network classifier was trained using the variables under study to predict when the concentration of fungal bioaerosols exceeded its average value (316 CFU/m^3^) to determine whether the analysed variables were sufficient to estimate the behaviour of fungal bioaerosols relative to their average value. The neural network provides insight into the extent to which the studied variables can explain the occurrence of high or low fungal bioaerosol concentrations, thereby complementing the initial analysis conducted with multivariate statistical models, which identify which of the studied variables are statistically related to the behaviour of fungal bioaerosol concentrations.

## 3 Results and Discussion

### 3.1 Fungal Species: Diversity, Distribution and Ecological Roles

This study analysed the concentrations of various fungal species present in bioaerosols at four monitoring sites in the metropolitan area of Barranquilla: three urbanised areas, Peace Square (S1), Barranquilla Riverwalk (S2), Puerto Colombia (S3), and one non‐urbanised area, Galapa (S4). A total of 10 viable fungal species were identified, including *Aspergillus niger*, *Aspergillus nidulans*, *Aspergillus fumigatus* , *Aspergillus terreus, Aspergillus flavus*, *Aspergillus versicolor*, *Aspergillus* sp., *Penicillium* sp., *Penicillium chrysogenum* and *F. oxysporum* (Table 1). Interestingly, the presence of these genera has been reported in various regions around the world, such as in India in both indoor and outdoor environments (Jabeen et al. 2023), as well as in the indoor and outdoor air of veterinary clinics in Iran (Mosalaei et al. 2021) and in hospitals in Tehran. However, studies conducted in high‐rise apartment buildings in Korea, both in indoor and outdoor environments, did not detect the *Fusarium* genus (Bolookat et al. 2018; Lee and Jo 2006). This absence may be attributed to differences in environmental conditions, such as humidity, temperature, and air circulation, which influence fungal growth and dispersion. Additionally, variations in sampling locations (e.g., urban vs. rural, high‐rise vs. ground‐level settings), seasonal differences during sampling, and the specific identification methods used (e.g., culture‐dependent vs. molecular techniques) may explain the disparity in findings.

**TABLE 1:** Concentration of identified fungal bioaerosol species.

Species	Peace Square (CFU/m^3^)	Barranquilla Riverwalk (CFU/m^3^)	Puerto Colombia (CFU/m^3^)	Galapa (CFU/m^3^)
*Aspergillus niger*	235.57 ± 21	294.46 ± 18	176.68 ± 15	285.40 ± 38
*Aspergillus nidulans*	244.63 ± 35	197.46 ± 35	239.78 ± 20	214.54 ± 18
*Aspergillus fumigatus*	176.68 ± 21	178.68 ± 41	198.76 ± 22	388.69 ± 53
*Aspergillus terreus*	176.68 ± 27	180.68 ± 12	2296.82 ± 102	
*Aspergillus flavus*		176.68 ± 15		176.68 ± 15
*Aspergillus versicolor*			235.57 ± 26	181.68 ± 20
*Aspergillus* sp.	264.19 ± 65	334.62 ± 52	277.64 ± 25	285.21 ± 35
*Penicillium* sp.	366.35 ± 52	377.09 ± 33	495.07 ± 51	339.97 ± 47
*Penicillium chrysogenum*	265.02 ± 51	198.76 ± 35	220.85 ± 18	294.46 ± 47
*Fusarium oxysporum*	176.68 ± 30	176.68 ± 12	197.46 ± 21	253.98 ± 60

*Note:* Errors are expressed as one times the standard deviation of the mean concentration calculated from the values obtained from the six replicates for each sampling day at the monitoring station.

In contrast, our study detected *F. oxysporum* in bioaerosols collected from all four monitoring sites, highlighting the distinct environmental characteristics of the Barranquilla metropolitan area. The presence of *Fusarium* in our samples underscores the potential role of local climate conditions, such as high humidity and temperature, and diverse microbial sources, in shaping fungal community composition. This finding aligns with other studies conducted in tropical and subtropical regions, where *Fusarium* is frequently reported in both indoor and outdoor air.

At site S1 (Peace Square), a notable presence of *A. nidulans* (244.63 ± 35 CFU/m^3^), *A. niger* (235.57 ± 21 CFU/m^3^), *Aspergillus sp*. (264.19 ± 65 CFU/m^3^), and *Penicillium sp*. (366.35 ± 52 CFU/m^3^) was observed (Table 1). These results suggest that this city area provides favourable conditions for the proliferation of these fungal species. Therefore, the presence of these species could be attributed to the proximity to a high‐traffic road (located 100 m away), the congregation of people at the bus stop, and the discharge of wastewater flowing through the stream channels. These factors could facilitate the dispersion of fungal bioaerosols from external sources. Similarly, at site S2 (Barranquilla Riverwalk), a higher concentration of *A. niger* (294.46 ± 18 CFU/m^3^) was recorded compared to site S1. High concentrations of *Penicillium* sp. (377.09 ± 33 CFU/m^3^) and *Aspergillus* sp. (334.62 ± 52 CFU/m^3^) were also detected. The presence of these species suggests that this area, heavily influenced by tourism activities and wastewater effluents, could be exposed to a more significant amount of fungal and/or bacterial bioaerosols carried by the Magdalena River. This occurs because contaminated rivers can transport organic material and microorganisms from other locations, facilitating their dispersion in the air as they reach the riverbanks. This phenomenon has been documented in previous studies, which have shown that the microbial load present in rivers can significantly contribute to the formation of bioaerosols in nearby areas (Morgado‐Gamero et al. 2025; Gong et al. 2020; Šantl‐Temkiv et al. 2020). In our study, the presence of *Penicillium sp*. could be related to the organic matter load and environmental conditions favourable for its development, as noted in the findings of Niazi et al. (2015).

On the other hand, at site S3 (Puerto Colombia), *A. terreus* exhibited an exceptionally high concentration (2296.82 ± 102 CFU/m^3^), significantly surpassing its levels at other sites (see Table 1 and Figure S1). This atypical concentration suggests the influence of a local factor, possibly related to the proximity of the Ciénaga de Mallorquín, an estuarine aquatic ecosystem whose ecological characteristics may provide favourable conditions for the proliferation and emission of marine –coastal aerosols rich in this species. Additionally, at this sampling site, a higher concentration of *Penicillium sp*. (495.07 ± 51 CFU/m^3^) and *Aspergillus versicolor* (235.57 ± 26 CFU/m^3^) were observed compared to the other sites studied. These high concentrations could also be associated with the influence of estuarine aerosols from the nearby Ciénaga de Mallorquín, reinforcing the hypothesis that the interaction between environmental factors and marine aerosols favours the proliferation of these fungi at the site.

At site S4 (Galapa), relevant concentrations of *Aspergillus niger* (285.40 ± 38 CFU/m^3^), *A. fumigatus* (388.69 ± 53 CFU/m^3^), *A. versicolor* (181.68 ± 20 CFU/m^3^), *Aspergillus sp*. (285.21 ± 35 CFU/m^3^), *Penicillium sp*. (339.97 ± 39 CFU/m^3^) and *P. chrysogenum* (294.46 ± 47 CFU/m^3^) were observed (Table 1). These values indicate a higher species diversity than the previous sites and could be related to the ecological richness of this natural environment, characterised by minimal anthropogenic impact. The highest microbial diversity in areas with minimal human activity is attributed to several key factors. Undisturbed natural environments provide more stable environmental conditions and a wide range of ecological niches, allowing for a greater variety of microbial species to coexist. In contrast, human activities such as agriculture, industry, urbanisation, and pollution disrupt the environmental conditions in which microbes live, reducing their diversity (Kumari et al. 2024). Soil disturbance, the use of fertilisers and pesticides, and changes in water chemistry (e.g., nutrient increase due to wastewater runoff) exert selective pressures that favour a few resistant species (van Rhijn et al. 2024). In contrast, areas with minimal human intervention, such as remote forests and pristine ecosystems, maintain more stable conditions, enabling the proliferation of a more significant number of microbial species (Li et al. 2020). These areas experience less influence from selective pressures, such as antibiotics and pollutants, which facilítate the coexistence of greater microbial diversity (Zheng et al. 2024).

Additionally, environments with minimal human intervention, such as tropical dry forests (similar to site S4), offer a greater variety of ecological niches that support the coexistence of specialised microbial communities with diverse ecological functions (Mo et al. 2024). Particular niches, such as high‐altitude areas and deep‐sea ventilation systems, are environments that accommodate specialised microbial communities, as observed in nature reserves like S4 (Ciccazzo et al. 2016).

The results obtained at each monitoring site consistently support the findings of previous studies that investigated fungal spores in the air, regardless of the collection methods used (Patel et al. 2018). The use of cultivation techniques (Fang et al. 2005), spore impaction (Ortega Rosas et al. 2020), and metagenomic exploration (Nicolaisen et al. 2017) has been documented, providing solid evidence on how environmental and anthropogenic characteristics in different urban areas can influence the distribution and abundance of fungal bioaerosols (Fatahinia et al. 2018; Kallawicha et al. 2015; Priyamvada et al. 2017). Studies conducted in tropical cities such as Fortaleza‐CE (Brazil), a coastal city, and in five outdoor areas of the city of São Luis (north, south, east, west and center) in the state of Maranhão, Brazil, as well as in Mexico City, which has a similar climate to Barranquilla, reported significant concentrations of *A. niger* and *A. flavus* in urban sites near anthropogenic pollution sources, such as roads with vehicular traffic and bus stops (Cajazeiras et al. 2022; Emygdio et al. 2018; del Carmen Calderón‐Ezquerro et al. 2021).

On the other hand, Rodríguez‐Gómez et al. (2020), in a region with environmental characteristics similar to S3 (Puerto Colombia), revealed high concentrations of *Aspergillus sp*. propagules in the Yucatán Peninsula–Playa Sisal, a coastal marine area affected by wastewater and human settlements. In line with these results, our study at site S3 found an exceptionally high concentration of *A. terreus* , the dominant species at this monitoring site (see Table 1 and Figure S1). The proximity to the Ciénaga de Mallorquín at site S3 and the influence of estuarine flora and marine aerosols in the Yucatán Peninsula–Playa Sisal could contribute to the abundance of *Aspergillus sp*. spores in these marine‐coastal environments (Eldin et al. 2022). Additionally, environmental pollution and the humidity conditions associated with the estuarine ecosystem could favour the growth and dispersion of *A. terreus* , as well as other species such as *A. nidulans* , *A. fumigatus* , *A. versicolor* and *Penicillium sp*., which also showed elevated concentrations at S3 (see Table 1 and Figure S1).

These observations align with the results obtained by Huertas et al. (2018), who evaluated bioaerosols in a tropical coastal city, specifically Cartagena de Indias, Colombia. Their study revealed that marine‐coastal environments affected by anthropogenic activities showed high concentrations of *Aspergillus sp*. and *Penicillium sp*., which support the presence of these fungal species at site S3, an area close to marine influence and also impacted by human activities. An important observation is that the genera *Alternaria sp*. and *Cladosporium sp*. were not reported, which were mentioned in previous studies conducted in Barranquilla in 2008 and 2019 using non‐cultivable methods, such as spore counting (Cepeda et al. 2018). This difference highlights the importance of using more than one approach to assess airborne fungi, particularly in terms of diversity, as some species may be difficult to cultivate under laboratory conditions (Jiménez López and Porras Duran 2019).

Finally, the results obtained at site S4 (Galapa), a natural reserve of tropical dry forest, support and expand on the findings from studies on fungal bioaerosols in tropical forest areas in China and Brazil, as highlighted by studies conducted by Zhang et al. (2010) and Barbosa et al. (2022), where elevated concentrations of *A. flavus* , *A. versicolor* and *P. chrysogenum* were reported. Similarly, our study recorded high concentrations of *A. flavus* (176.68 ± 15 CFU/m^3^), *A. versicolor* (181.68 ± 20 CFU/m^3^) and *P. chrysogenum* (294.46 ± 47 CFU/m^3^) at S4 (see Table 1 and Figure S1). This pattern of fungal species presence in different tropical regions reinforces the idea that certain groups of fungal species may be shared and dominant in forest ecosystems of this nature. Furthermore, a key aspect of S4 is its ecological richness and the limited anthropogenic activity in the area where the cascade impactor was placed. The absence of significant disturbances caused by human activities, such as deforestation or urbanisation, could provide more stable and favourable environmental conditions for the development and persistence of various airborne fungal species.

### 3.2 Concentration of Fungal Bioaerosols: Spatial and Temporal Trends

**FIGURE 2:**
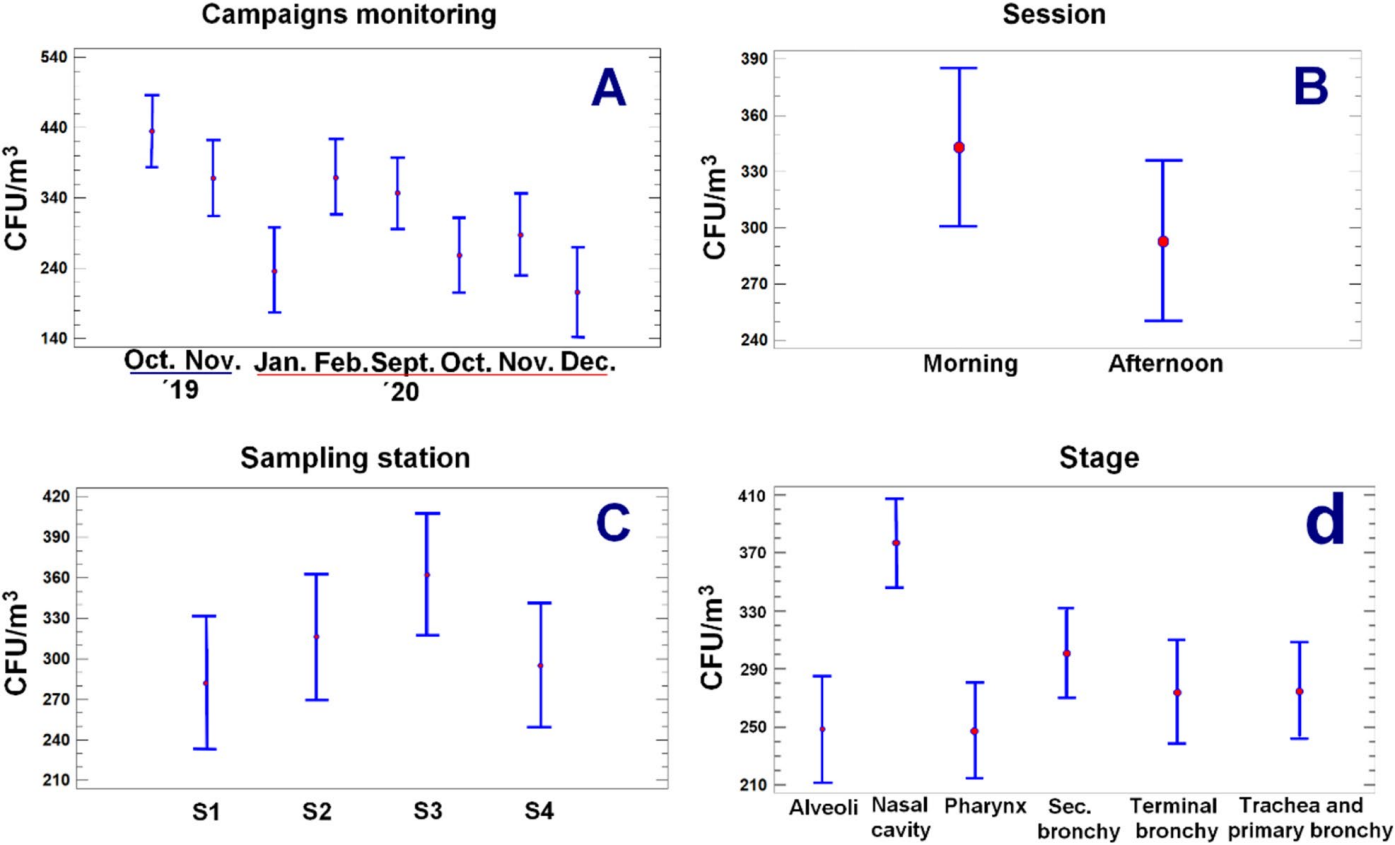
Type III sum of squares model based on general linear models, showcasing means and 95% LSD intervals for campaigns monitoring (a), session (b), sampling stations (c) and stages (d).

The concentration of fungal bioaerosols at all sites (S1, S2, S3 and S4) throughout the dry and rainy seasons is shown in detail in Table 2. Measurements were collected during the morning (M) and afternoon (A) to understand fungal bioaerosols' temporal and spatial fluctuations fully. All parameters studied significantly impact the concentration of fungal bioaerosols, with *p* values < 0.05, according to the analysis employing the type III sum of squares model (see Tables 2 and 3, Figure 2 and Table S1). This suggests that varied monitoring campaigns during both the dry and rainy seasons, as well as the sampling site and hour (morning or afternoon), have a considerable impact on fungal bioaerosol concentration (see Figure 2).

**TABLE 2:** The concentration of fungal bioaerosols (CFU/m^3^) during the dry and rainy seasons.

Sampling station		Campaigns Monitoring								Average
		Oct. 19^a^	Nov. 19^a^	Jan. 20^b^	Feb. 20^b^	Sept. 20^b^	Oct. 20^b^	Nov. 20^a^	Dec. 20^a^	
S1—Peace square	M	402.43 ± 95.20	224.86 ± 36.00	190.27 ± 51	294.46 ± 45.40	441.70 ± 58.30	206.12 ± 14.5	176.68 ± 12.2	206.12 ± 18.6	269.86 ± 33.12
	A	345.33 ± 54	265.02 ± 51.20	270.21 ± 32.02	228.64 ± 25.50	344.94 ± 25.45	229.68 ± 27.80	235.57 ± 20.50	176.68 ± 15.44	253.40 ± 25.06
S2—Barranquilla Riverwalk	M	388.69 ± 42	297.56 ± 35.65	338.63 ± 29.54	294.46 ± 22.30	380.13 ± 32.30	227.16 ± 51.20	378.60 ± 41.20	176.68 ± 21.61	315.93 ± 32.06
	A	390.55 ± 58.04	239.78 ± 29.04	253.98 ± 27.50	309.19 ± 35.60	325.46 ± 65.30	198.76 ± 41.20	176.68 ± 21.30	206.12 ± 29.42	273.61 ± 36.80
S3—Puerto Colombia	M	307.55 ± 51.6	247.35 ± 27.60	268.55 ± 30.40	280.61 ± 30.50	274.83 ± 55.30	309.19 ± 33.60	223.79 ± 42.10	189.30 ± 21.55	262.52 ± 33.04
	A	328.12 ± 25	229.68 ± 22.40	738.84 ± 89.20	307.6 ± 48.10	757.19 ± 123.56	196.31 ± 51.20	208.80 ± 35.60	176.68 ± 41.45	360.88 ± 51.33
S4—Galapa	M	377.64 ± 23.4	276.68 ± 18.90	269.81 ± 24.36	346.57 ± 32.52	391.22 ± 36.10	257.16 ± 42.10	318.02 ± 45.64	285.09 ± 27.62	312.85 ± 12.30
	A	329.26 ± 24.7	201.92 ± 18.54	250.29 ± 54.40	360.42 ± 45.90	288.79 ± 98.41	256.99 ± 26.50	256.18 ± 54.63	176.68 ± 30.51	264.13 ± 17.08

*Note:* Errors are expressed as one times the standard deviation of the mean concentration calculated from the values obtained from the six replicates for each sampling day at the monitoring station.

Abbreviations: A: afternoon; M: morning.

^a^ Months affected by the rainy season.

^b^ Months affected by the dry season.

**TABLE 3:** Type III sum of squares model for fungal bioaerosol concentrations concerning the factors.

Factor	Sum of squares	Df	Mean square	F‐ratio	*p*
Campaign	4.74 × 10^6^	7	676669.32	6.89	0.0000
Session (day)	617257.34	1	617257.52	6.28	0.0122
Monitoring station	934589.32	3	311530.78	3.17	0.0236
Stage	1.92 × 10^6^	5	383694.85	3.90	0.0016
Species	5.61 × 10^6^	10	467152.20	4.75	0.0000
Temperature	549083.21	1	549083.56	5.59	0.0181
Residual	1.05 × 10^6^	1070	98280.67		
Total	1.22 × 10^8^	1099			

In all monitoring sites, throughout the dry and the wet seasons, the results show that fungal bioaerosol concentrations are often higher in the morning than in the afternoon (*p* value = 0.0122, Figure 2B and Table 3). Compared to the afternoon readings of 253.40 ± 25.06 CFU/m^3^ and 273.61 ± 36.81 CFU/m^3^ in sites S1 (Peace Square) and S2 (Barranquilla Riverwalk), the average concentrations were higher in the morning with values of 269.86 ± 33.13 CFU/m^3^ and 315.93 ± 32.06 CFU/m^3^, respectively (see Table 2). The concentrations in site S4 (Galapa) were also considerably higher in the morning sampling site (312.85 ± 12.30 CFU/m^3^) than in the afternoon sampling site (264.13 ± 17.08 CFU/m^3^. The temporal and spatial variability observed in fungal bioaerosol concentrations is influenced by meteorological conditions and human and animal activities that change throughout the day and region. As described by Morgado‐Gamero et al. (2025) and Sajjad et al. (2023), the morning and afternoon periods are characterised by varying meteorological conditions and generate different, even contrasting, sampling conditions.

Additionally, certain activities can increase the production and dispersion of bioaerosols, such as increased human activity (including transportation to schools, work areas, and parks, among others), animal activity and wind speed. These activities are more frequent during morning hours when people are more on the move. However, these activities depend on economic and sociocultural components, which vary significantly within our population.

Sunlight intensity, which increases throughout the day, is another factor that may explain these differences. In tropical regions like Barranquilla, where intense sunlight and high UV radiation are present year‐round, afternoon exposure to UV radiation can significantly reduce the viability and concentration of fungal spores (Braga et al. 2015; Ghajar et al. 2006; Rotem and Aust 1991). This reduction likely explains the observed pattern of higher fungal bioaerosol concentrations in the morning compared to the afternoon. Overnight, fungal spores may accumulate in the atmosphere due to lower temperatures, minimal air movement, and reduced UV exposure, leading to higher concentrations in the morning. As the day progresses, increased sunlight and UV radiation contribute to the degradation of fungal spores, alongside meteorological factors such as higher wind speeds and reduced humidity, which can further impact their viability and dispersal. This diurnal variability and human activities such as commuting and outdoor movement likely contribute to the observed temporal differences in fungal bioaerosol concentrations between morning and afternoon in Barranquilla.

An exception is seen at site S3 (Puerto Colombia), where the average afternoon concentration unexpectedly increased to 360.88 ± 51.33 CFU/m^3^ from 262.52 ± 33.04 CFU/m^3^ in the morning (see Table 2). However, some sites presented a different pattern. Contrary to the usual pattern, concentrations were typically higher in the mornings during the dry and rainy seasons. Based on the findings of this study, the morning hours have a more significant concentration of fungal bioaerosols due to more favourable environmental conditions for the release and spread of fungal spores. This theory is supported by earlier research, which demonstrates that throughout the night, temperatures drop, and relative humidity rises in the study area (Morgado‐Gamero et al. 2021), encouraging the buildup of spores on surfaces and in the soil (Jones and Harrison 2004). These higher aerosol peaks happen in the morning when the temperature rises and coincide with the start of the day. These sites corresponded to the peri‐urban sites. Notably, two outlier values in S3 (Puerto Colombia) were found to impact the bioaerosol average concentrations. A concentration of 738.84 ± 89.20 CFU/m^3^ was observed in January, during the dry season, over three times greater than the morning value (see Table 2).

Similarly, in site S4 (Galapa), the concentrations during the morning were markedly higher, with a value of 312.85 ± 12.30 CFU/m^3^, compared to 264.13 ± 17.08 CFU/m^3^ during the afternoon (see Table 2). Similarly, at the start of the rainy season, 757.19 ± 123.56 CFU/m^3^ was reached in September 2020, about three times greater than the morning concentration (see Table 2). The Vía Isla Salamanca Natural Park, which is 2.5 km away from site S3 (Puerto Colombia), was affected by forest fires, particularly during the dry season, which coincided with the campaigns in S3 during the third and fifth months. These fires could be attributed, at least in part, to the increasing concentration. Moreover, the overall pattern was observed in Figure 2B, where the afternoon mean concentrations were higher than the morning mean ones.

On the other hand, fungal bioaerosol concentrations tend to decline in the afternoon, most likely due to the soil and atmosphere warming, which results in reduced atmospheric stability and more vertical mixing (Seinfeld and Pandis 2016). As a result, spores are dispersed, and concentrations are diluted (Adhikari et al. 2006). Additionally, according to certain studies, some fungal species may be more susceptible to variations in temperature and humidity in the afternoon, which could impact their spore release and, as a result, their concentration in the atmosphere (Heo et al. 2023; Jones and Harrison 2004). This study discovered a significant relationship between bioaerosol concentrations and temperature differences between morning and afternoon sample sites (*p* value = 0.0181, Table 3). These results emphasise the significance of considering these aspects when researching the dynamics of fungal bioaerosols in the atmosphere and support their influence on diurnal ambient conditions.

Significant variations in fungal bioaerosol concentrations were found between the sampling sites (*p* value = 0.0236, Figure 2C, Table 3). For instance, at site S1 (Peace Square), concentrations in the sampling locations and campaigns range from roughly 176.68 ± 12.2 to 441.70 ± 58.3 CFU/m^3^. On the other hand, concentrations at Puerto Colombia site‐S3 fluctuate between 176.68 ± 41.5 and 757.19 ± 123.56 CFU/m^3^ (see Table 2). These differences might be explained by particular environmental elements present at each site. Intense human activity, such as motor vehicle traffic, public demonstrations, and residential buildings surrounding site‐S1 (Peace Square) in the city's Historic Center (see Figure 1). These elements may increase the amount of fungal bioaerosols released into the environment. Barranquilla Riverwalk (S2) is close to the shores of the Magdalena River, which receives various effluents from other municipalities, deteriorating its water quality and potentially increasing the microbial load in the area, affecting fungal bioaerosol concentrations (see Figure 1). Additionally, the influence of tourist activities and port operations could also contribute to the presence of fungal bioaerosols at this site.

The Ciénaga de Mallorquín, an estuarine aquatic habitat impacted by sewage pollution and human settlements, lies 800 m from site S3 (Puerto Colombia). These environmental factors may affect the survival and dispersion of microbial bioaerosols, including fungi, in the air at this location. The building of residential units is also relatively close (200 m) to S3, and its impact on the surrounding vegetation, production of dust and suspended particles, and buildup of organic matter may make it a rich source of fungal bioaerosols. Site S4 (Galapa) is in a tropical dry forest natural reserve with much ecological variety. This site might help the local ecosystem contain various fungal bioaerosols.

Additionally, all sampling sites show a definite difference between the dry and wet seasons (*p* values of 0.0000 and 0.0122, respectively, Table 3). Compared to the rainy season, fungal bioaerosol concentrations are typically lower during the dry season (see Tables 2 and 3). This variation can be linked to the research area's rainy season climate, which encourages the dispersal and release of fungus spores into the atmosphere. Each season's unique climatic and environmental variables can be used to explain differences in fungal bioaerosol concentration between the dry and wet seasons (Amarloei et al. 2020; Kowalski and Pastuszka 2018).

Fungi and other microorganisms have a greater ability to grow and disperse during the rainy season due to increased humidity and water availability. These conditions favour the flourishing and spread of fungi and their spores. In this regard, the results obtained from our research align with studies conducted in open urban areas with similar meteorological conditions, which have reported significantly higher concentrations of fungal bioaerosols during the rainy season compared to the dry season (Morgado‐Gamero et al. 2025; Zhong et al. 2016; Kang et al. 2015).

### 3.3 The Aerodynamics of Fungal Bioaerosol Inhalation: Size‐Dependent Penetration and Possible Implications for Human Respiratory Health

**FIGURE 3:**
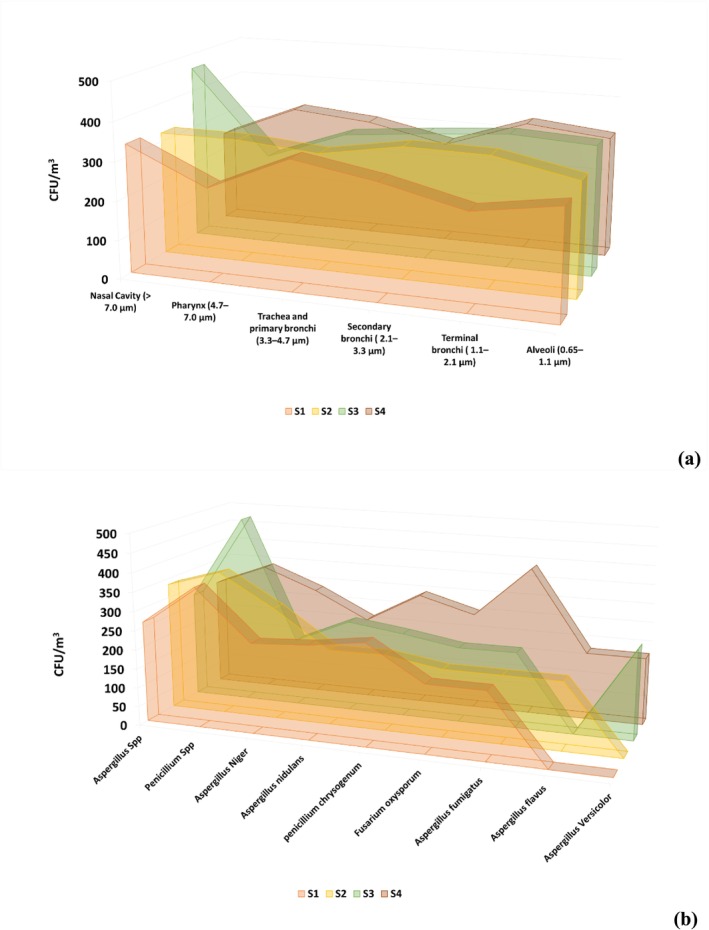
Mean concentration of fungal bioaerosol by sampling site and size range (a); total mean concentration for each species and aerodynamic size (b); Size ranges in these diameters: > 7.0 μm (Nasal Cavity), 4.7–7.0 μm (Pharynx), 3.3–4.7 μm (Trachea and primary bronchi), 2.1–3.3 μm (Secondary bronchi), 1.1–2.1 μm (Terminal bronchi) and 0.65–1.1 μm (Alveoli).

Figures 2 and 3 show the size distribution of total mean fungal bioaerosols in this study regarding the human respiratory system. We found statistical differences in concentration between the aerodynamic sizes with a *p* value of 0.0016 (see Table 3). Figure 3A shows that the coarse fraction (> 4.7 μm) was the dominant size fraction for the total mean concentration of this study; bioaerosols of this aerodynamic size can deposit in the upper respiratory system, such as the nasal cavity and pharynx. Another dominant fraction was the aerodynamic size of particles between 3.3 and 2.1 μm; these particles can deposit in the secondary bronchi, while the fine fraction (< 2.1 μm) reported lower but considerable results of the particles that can reach the terminal bronchi and alveoli affecting the lower respiratory human system. Depending on the fungal species and the receptor, Figure 2D shows species per aerodynamic size; our results reported the presence of species such as *Aspergillus* sp., *A. fumigatus* , *A. niger* , *Penicillium* sp. and *Fusarium* sp. in all the aerodynamic sizes, even those particles able to penetrate until the alveoli. These species have been associated with being opportunistic, pathogenic, or allergenic and represent a risk to human health depending on the receptor (Grewling et al. 2023). Because spores are airborne and microscopic, they are unavoidably inhaled (Anees‐Hill et al. 2022), but immunocompetent hosts can eliminate them by innate immune mechanisms. However, with increases in the number of immunosuppressed patients, there has been a dramatic increase in severe invasive aspergillosis (Grewling et al. 2023). For example, *A. fumigatus* drives allergic fungal airway disease and IgE sensitisation, and *Penicillium* sp. *and Aspergillus* sp. have 35 and 13 allergens described, respectively. *Aspergillus* genera can cause allergies, respiratory infections, asthma, and lung disease (chronic pulmonary aspergillus) (Qi et al. 2020).

Figures 2C,D and 3A show the aerodynamic size per sampling site; in this study, all the sampling areas, urban or non‐urban, reported fungal bioaerosols in all the aerodynamic sizes; however, metropolitan areas (S1, S2 and S3) presented higher concentrations in the nasal cavity and secondary bronchi with lower concentrations in the terminal bronchi and alveoli. We observed an increase in the nasal cavity concentration in Puerto Colombia (S3); the high concentration of *A. terreus* can explain this (see Figure S1 and Table 1); in contrast, this species was not found in the non‐urbanised area, Galapa (S4) (see Table 1). *Aspergillus* genera, *Penicillium* and *Fusarium* reported higher concentrations in S1, S2 and S3; these genera have been highly associated with urban areas (Qi et al. 2020). In the non‐urban area, the fungal aerosols did not show drastic differences between concentrations of the aerodynamic sizes that represent the human respiratory system. Due to anthropogenic activities, bioaerosols in urban areas (S1, S2 and S3) might differ significantly from non‐urban areas (S4); our results showed statistical differences between the sampling sites with a *p* value 0,0236 (see Figure 2C and Table 3).

### 3.4 Modelling Fungal Distribution: An Artificial Neural Network Approach

**FIGURE 4:**
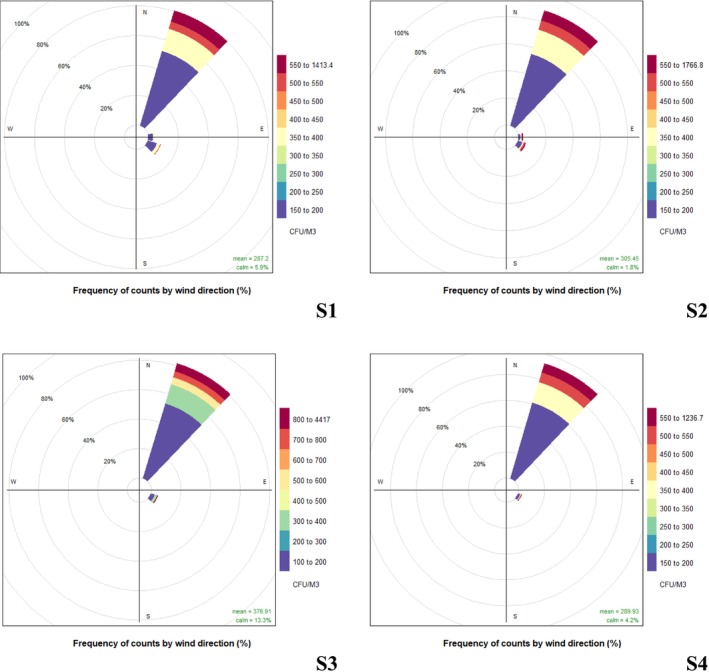
The wind rose pollution of all sampling sites, urbanised areas: (A) S1 Peace Square de la Paz. (B) S2 Barranquilla Riverwalk del Rio. (C) S3 Puerto Colombia and non‐urbanised area: (D) Galapa.

Figure 4 displays the pollution rose for each site. It is evident that, for most sites, the concentrations predominantly fell within the ranges of 150–200 CFU/m^3^ and 340–400 CFU/m^3^. Notably, this distribution was held for all sites except for S3 (Puerto Colombia). This observation underscores that the minimum concentration value recorded across the study area was 150 CFU/m^3^. As previously discussed, Figure 4 reaffirms that S3 exhibited the highest pollutant concentrations, up to 4417 CFU/m^3^. The second most frequent values consistently fell within the 300–350 CFU/m^3^ range across all sites. At first glance, it may be seen that almost all airborne fungi are coming from the NE direction, with a minimal contribution from the E and SE directions.

**FIGURE 5:**
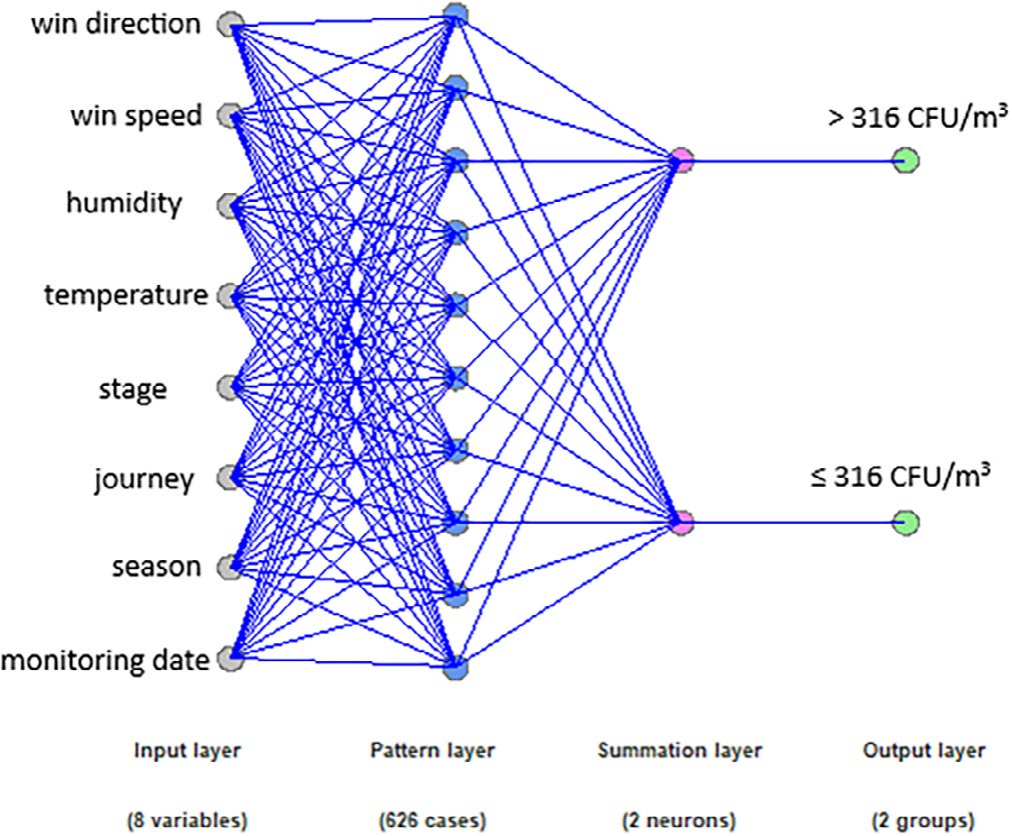
Schematic representation of multicap perceptron neural network used to classify cases when the concentration of the fungal bioaerosols exceeded their lowest value (316 CFU/m^3^). The correct forecasting capacity of the network was 76.87% based on the parameters of this study.

A Bayesian neural network was trained using the variables from this study to predict when the concentration of fungal bioaerosols exceeded its average value (316 CFU/m^3^) to determine whether the variables were sufficient to estimate when the fungal bioaerosol reached a value surpassing its average (see Tables S2–S4). The correct prediction capacity of the network on the validation set was 76.87%, based on parameters such as the season (sampling campaign), time of day (morning or afternoon), Andersen impactor stage (aerodynamic size), temperature, humidity, wind speed and direction. This indicates that these parameters provide relevant and sufficient information to make an approximate estimation of whether the bioaerosol would reach high concentrations. However, to improve the network's prediction capacity, more relevant parameters influencing the occurrence of high fungal bioaerosol concentrations in urban areas should be included (see Figure 5, Tables S2 and S3).

### 3.5 Fungal Bioaerosols Concentrations Pre‐ and Post‐Lockdown

Since the monitoring campaigns were carried out before and after the COVID‐19 isolation period, a multifactorial ANOVA was performed to analyse the results (see Table S5). The analysis showed that the fungal concentration (CFU/m^3^) had statistically significant results for the main effects of the factors ‘confinement’ and ‘impactor stage’, as well as a significant interaction between these factors. Specifically, fungal concentrations significantly differed before and after the isolation period (*p* < 0.0001 in Table S5).

These results reflect notable changes in airborne fungal dispersion dynamics, influenced by the restrictive measures implemented during the confinement. The pandemic led to a significant reduction in leisure activities, travel, and general well‐being, which reduced outdoor activities and posed challenges to the mental and physical health of those who prefer natural environments. Nevertheless, this period also had positive effects, such as decreased pollution, reduced bioaerosol generation, and a calmer atmosphere with cleaner and quieter beaches, parks, and urban areas (Konishi et al. 2024). This situation likely contributed to reduced airborne fungal dispersion during the isolation period.

On the other hand, the multifactorial analysis also indicated that the factor ‘impactor stage’ (*p* = 0.0286, Table S5) had a significant effect on fungal concentrations, which varied across the different stages of the cascade impactor. This variation may be related to the impactor's ability to classify particles based on their size, which affects the amount of fungi deposited at each stage. Differences in concentration between stages are expected, as aerosols of different sizes tend to distribute differently depending on activity levels and atmospheric conditions at the monitored sites, as observed in the collected data (see Tables 1 and 2, Figure 3).

Between 2019 and 2021, covering the periods before, during, and after COVID‐19, environmental changes such as variations in noise and air pollution were influenced by the progressive relaxation of pandemic restrictions. A steady increase in vehicular traffic characterised this transition, with more people engaging in physical activities and sports in public spaces and the reopening of factories, businesses, and airports (Schöttl et al. 2022; Madhwal et al. 2020a; Madhwal et al. 2020b). These post‐confinement conditions once again favoured the emission of bioaerosols, leading to a gradual increase in fungal concentrations as the city returned to normal.

The significant interaction between the factors ‘confinement’ and ‘impactor stage’ (*p* < 0.0001, Table S5) indicates that the effect of isolation was not uniform across all impactor stages. Fisher's LSD test showed that the most significant difference in fungal concentration was observed in the nasal cavity before isolation compared to the post‐confinement period (see Figure S1). Before isolation, the conditions favoured a higher concentration of fungi in this region, whereas confinement measures likely reduced their dispersion. However, no significant differences were found in the other impactor stages, as the LSD intervals overlapped, suggesting that the reduction in fungal concentration was not homogeneous across all classification phases of the impactor.

Our results demonstrate that pre‐pandemic, the concentrations of bioaerosols associated with fungal contamination were higher compared to the isolation period. As restrictions were lifted, the city gradually began to return to its pre‐pandemic state, reflected in an increase in airborne fungal concentrations. It should be noted that the Colombian government decreed the isolation period in our country, which began on March 20, 2020, while the complete reopening of the country, without restrictions, started on September 1 of the same year. However, persistent fear among the population delayed the return to normality, making the transition gradual.

## 4 Conclusions

This study identified 10 fungal species in bioaerosols across four sites in the Barranquilla metropolitan area, including *A. niger*, *A. nidulans* , *A. fumigatus* , *A. terreus* , *A. flavus* , *A. versicolor* , *Aspergillus* sp., *Penicillium* sp., *P. chrysogenum* and *F. oxysporum*.

The distribution of these species varied across sites, with urban areas (Peace Square, Barranquilla Riverwalk, and Puerto Colombia) exhibiting higher concentrations than the non‐urban area (Galapa). Notably, Peace Square, located near a high‐traffic road and wastewater channels, showed a significant presence of *A. nidulans* (244 CFU/m^3^), *A. niger* (235 CFU/m^3^), *Aspergillus* sp. (264 CFU/m^3^), and *Penicillium* sp. (366 CFU/m^3^), suggesting these factors contribute to fungal bioaerosol dispersion. Barranquilla Riverwalk, influenced by tourism and wastewater effluents, had a high concentration of *A. niger* (294 CFU/m^3^), *Penicillium* sp. (377 CFU/m^3^) and *Aspergillus* sp. (334 CFU/m^3^), potentially linked to the microbial load carried by the Magdalena River. Puerto Colombia, near the Ciénaga de Mallorquín, exhibited an exceptionally high concentration of *A. terreus* , possibly due to the estuarine ecosystem's influence. Galapa, with minimal anthropogenic impact, displayed the highest fungal diversity, including *A. niger* , *A. fumigatus* , *A. versicolor* , *Aspergillus* sp., *Penicillium* sp. and *P. chrysogenum*, reflecting the ecological richness of the undisturbed natural environment.

Fungal bioaerosol concentrations were consistently higher in the morning (~340 CFU/m^3^) than in the afternoon (~300 CFU/m^3^) across all sites, likely due to the favourable conditions for spore release and dispersion created by lower temperatures and higher humidity during the night. This pattern was consistent across both dry and rainy seasons. However, Puerto Colombia showed an atypical increase in the afternoon during the dry season, possibly due to forest fires in the nearby Vía Isla Salamanca Natural Park.

The study also found that the aerodynamic size of fungal particles significantly influences their potential impact on human health. Coarser particles (> 4.7 μm), which deposit in the upper respiratory tract, were dominant, especially in urban areas. However, finer particles capable of reaching the alveoli were also present, posing a potential risk for immunocompromised individuals.

A Bayesian neural network model, incorporating factors such as season, time of day, and meteorological conditions, successfully predicted high fungal bioaerosol concentrations with 76.87% accuracy. This suggests that these variables are informative for understanding fungal bioaerosol behaviour, but additional parameters may be needed to improve predictive accuracy, especially in urban areas with high concentrations.

Finally, the study found that fungal bioaerosol concentrations were significantly affected by the COVID‐19 lockdown, with lower concentrations observed during the lockdown period compared to pre‐ and post‐lockdown periods. This highlights the impact of human activity on bioaerosol dispersion.

## Author Contributions

**Euler Gallego‐Cartagena:** conceptualization, investigation, validation, supervision, writing – review and editing. **Wendy Morgado‐Gamero:** investigation, validation, methodology, writing – original draft. **Iuleder de Moya‐Hernández:** investigation, software. **Carlos Díaz‐Uribe:** formal analysis, data curation, conceptualization. **Alexander Parody:** software, formal analysis, visualization. **Héctor Morillas:** methodology, visualization, data curation. **Brayan Bayona‐Pacheco:** visualization, data curation, conceptualization. **Gabrielle Pellegrin:** validation, data curation. **Dayana Agudelo‐Castañeda:** conceptualization, validation.

## Ethics Statement

The authors have nothing to report.

## Conflicts of Interest

The authors declare no conflicts of interest.

## Supporting information


**Data S1.** Supporting Information.

## Data Availability

The data that support the findings of this study are available from the corresponding author upon reasonable request.

## References

[emi470078-bib-0001] Adhikari, A. , T. Reponen , S. A. Grinshpun , D. Martuzevicius , and G. LeMasters . 2006. “Correlation of Ambient Inhalable Bioaerosols With Particulate Matter and Ozone: A Two‐Year Study.” Environmental Pollution 140, no. 1: 16–28. 16183184 10.1016/j.envpol.2005.07.004

[emi470078-bib-0002] Agudelo‐Castañeda, D. , F. de Paoli , W. B. Morgado‐Gamero , et al. 2020. “Assessment of the NO_2_ Distribution and Relationship With Traffic Load in the Caribbean Coastal City.” Science of the Total Environment 720: 137675. 32325599 10.1016/j.scitotenv.2020.137675

[emi470078-bib-0003] Agudelo‐Castañeda, D. M. , E. Calesso Teixeira , L. Alves , J. A. Fernández‐Niño , and L. A. Rodríguez‐Villamizar . 2019. “Monthly‐Term Associations Between Air Pollutants and Respiratory Morbidity in South Brazil 2013–2016: A Multi‐City, Time‐Series Analysis.” International Journal of Environmental Research and Public Health 16, no. 20: 1–13. 10.3390/ijerph16203787 PMC6843508 31600878

[emi470078-bib-0004] Amarillo, A. C. , and H. A. Carreras . 2012. “The Effect of Airborne Particles and Weather Conditions on Pediatric Respiratory Infections in Cordoba, Argentine.” Environmental Pollution 170: 217–221. 22835501 10.1016/j.envpol.2012.07.005

[emi470078-bib-0005] Amarloei, A. , M. Fazlzadeh , A. J. Jafari , A. Zarei , and S. Mazloomi . 2020. “Particulate Matters and Bioaerosols During Middle East Dust Storms Events in Ilam, Iran.” Microchemical Journal 152: 104280.

[emi470078-bib-0006] Amaya Díaz, L. V. , J. F. López Soto , M. A. Orcasita Almarales , A. S. Ochoa Arrieta , C. D. Pacheco Díaz , and M. J. Padrón Echenique . 2021. “Caracterización demográfica y algunos aspectos clínicos de interés en pacientes con tuberculosis pulmonar bajo vigilancia del Programa Mired Barranquilla 2020–2021.”

[emi470078-bib-0007] Anees‐Hill, S. , P. Douglas , C. H. Pashley , A. Hansell , and E. L. Marczylo . 2022. “A Systematic Review of Outdoor Airborne Fungal Spore Seasonality Across Europe and the Implications for Health.” Science of the Total Environment 818: 151716. 34800445 10.1016/j.scitotenv.2021.151716 PMC8919338

[emi470078-bib-0008] Awokola, B. I. , G. Okello , K. J. Mortimer , C. P. Jewell , A. Erhart , and S. Semple . 2020. “Measuring Air Quality for Advocacy in Africa (MA3): Feasibility and Practicality of Longitudinal Ambient PM_2.5_ Measurement Using Low‐Cost Sensors.” International Journal of Environmental Research and Public Health 17, no. 19: 7243. 33023037 10.3390/ijerph17197243 PMC7579047

[emi470078-bib-0009] Barbosa, C. G. , P. E. Taylor , M. O. Sá , et al. 2022. “Identification and Quantification of Giant Bioaerosol Particles Over the Amazon Rainforest.” Npj Climate and Atmospheric Science 5, no. 1: 73.

[emi470078-bib-0010] Betancur‐Otalvaro, J. P. , J. E. Estrada‐Pedrozo , Y. Pinillos‐Patiño , E. Prieto‐Suárez , and R. García‐Jiménez . 2023. “Determinantes de la hospitalización en pacientes con diagnóstico de bronquiolitis en Barranquilla, Colombia.” Revista de Salud Pública 22: 589–593. 10.15446/rsap.V22n6.86074 36753076

[emi470078-bib-0011] Bolookat, F. , M. S. Hassanvand , S. Faridi , M. Hadei , M. Rahmatinia , and M. Alimohammadi . 2018. “Assessment of Bioaerosol Particle Characteristics at Different Hospital Wards and Operating Theaters: A Case Study in Tehran.” MethodsX 5: 1588–1596. 30622921 10.1016/j.mex.2018.11.021 PMC6313819

[emi470078-bib-0012] Bongomin, F. , S. Gago , R. O. Oladele , and D. W. Denning . 2017. “Global and Multi‐National Prevalence of Fungal Diseases—Estimate Precision.” Journal of Fungi 3, no. 4: 57. 29371573 10.3390/jof3040057 PMC5753159

[emi470078-bib-0013] Braga, G. U. L. , D. E. N. Rangel , É. K. K. Fernandes , S. D. Flint , and D. W. Roberts . 2015. “Molecular and Physiological Effects of Environmental UV Radiation on Fungal Conidia.” Current Genetics 61, no. 3: 405–425. 10.1007/s00294-015-0483-0. 25824285

[emi470078-bib-0014] Bruni, E. , G. Simonetti , B. Bovone , et al. 2020. “Evaluation of Bioaerosol Bacterial Components of a Wastewater Treatment Plant Through an Integrated Approach and In Vivo Assessment.” International Journal of Environmental Research and Public Health 17, no. 1: 273. 10.3390/ijerph17010273 PMC6981557 31906026

[emi470078-bib-0015] Bui, L. T. , H. T. N. Lai , and P. H. Nguyen . 2023. “Benefits of Short‐Term Premature Mortality Reduction Attributed to PM_2.5_ Pollution: A Case Study in Long an Province, Vietnam.” Archives of Environmental Contamination and Toxicology 85: 245–262. 37468649 10.1007/s00244-023-01012-2

[emi470078-bib-0016] Cajazeiras, Í. M. P. , F. C. T. D. Carvalho , J. O. Abreu , et al. 2022. “Microbial Content of Bioaerosols in Outdoor Urban Recreation Areas of an Atlantic Coastal City (Fortaleza‐CE, Brazil).” Journal of Air Pollution and Health 7, no. 2: 205–216.

[emi470078-bib-0017] Cepeda, A. , S. Villalba , A. Parody , and M. Gamboa . 2018. “Calendario polínico de Barranquilla, Colombia, 2008–2013. En: Memorias del Congreso de la sociedad española de alergología e inmunología clínica, Valencia‐España,” p. 265.

[emi470078-bib-0018] Ciccazzo, S. , A. Esposito , L. Borruso , and L. Brusetti . 2016. “Microbial Communities and Primary Succession in High Altitude Mountain Environments.” Annals of Microbiology 66: 43–60.

[emi470078-bib-0019] Cohen, A. J. , M. Brauer , R. Burnett , et al. 2017. “Estimates and 25‐Year Trends of the Global Burden of Disease Attributable to Ambient Air Pollution: An Analysis of Data From the Global Burden of Diseases Study 2015.” Lancet 389, no. 10082: 1907–1918. 28408086 10.1016/S0140-6736(17)30505-6 PMC5439030

[emi470078-bib-0020] DANE . 2021. “National Population and Housing Census.” https://microdatos.dane.gov.co/index.php/catalog/643/study‐description.

[emi470078-bib-0021] D'Arcy, N. , E. Cloutman‐Green , K. M. Lai , D. Margaritis , N. Klein , and D. A. Spratt . 2014. “Potential Exposure of Children to Environmental Microorganisms in Indoor Healthcare and Educational Settings.” Indoor and Built Environment 23, no. 3: 467–473.

[emi470078-bib-0022] del Carmen Calderón‐Ezquerro, M. , N. Serrano‐Silva , and C. Brunner‐Mendoza . 2021. “Aerobiological Study of Bacterial and Fungal Community Composition in the Atmosphere of Mexico City Throughout an Annual Cycle.” Environmental Pollution 278: 116858. 33740598 10.1016/j.envpol.2021.116858

[emi470078-bib-0023] Denham, S. T. , M. A. Wambaugh , and J. C. Brown . 2019. “How Environmental fungi Cause a Range of Clinical Outcomes in Susceptible Hosts.” Journal of Molecular Biology 431, no. 16: 2982–3009. 31078554 10.1016/j.jmb.2019.05.003 PMC6646061

[emi470078-bib-0024] Denning, D. W. , and E. F. Morgan . 2022. “Quantifying Deaths From Aspergillosis in HIV Positive People.” Journal of Fungi 8, no. 11: 1131. 36354898 10.3390/jof8111131 PMC9693143

[emi470078-bib-0025] Després, V. , J. A. Huffman , S. M. Burrows , et al. 2012. “Primary Biological Aerosol Particles in the Atmosphere: A Review.” Tellus B: Chemical and Physical Meteorology 64, no. 1: 15598.

[emi470078-bib-0026] Eldin, A. M. , S. F. S. Al‐Sharnouby , K. I. M. ElGabry , and A. I. Ramadan . 2022. “Aspergillus Terreus, *Penicillium* Sp. and *Bacillus* Sp. Isolated From Mangrove Soil Having Laccase and Peroxidase Role in Depolymerization of Polyethylene Bags.” Process Biochemistry 118: 215–226.

[emi470078-bib-0027] Emygdio, A. P. M. , M. de Fátima Andrade , F. L. T. Gonçalves , G. Engling , R. H. de Souza Zanetti , and P. Kumar . 2018. “Biomarkers as Indicators of Fungal Biomass in the Atmosphere of São Paulo, Brazil.” Science of the Total Environment 612: 809–821. 28881304 10.1016/j.scitotenv.2017.08.153

[emi470078-bib-0028] Esch, R. E. , J. A. Bernstein , and H. M. Vijay . 2020. “Fungal Allergens.” In Allergens and Allergen Immunotherapy: Subcutaneous, Sublingual, and Oral, edited by R. F. Lockey and D. K. Ledford , 6th ed., 189–211. CRC Press/Taylor and Francis Group.

[emi470078-bib-0029] Fang, Z. , Z. Ouyang , L. Hu , X. Wang , H. Zheng , and X. Lin . 2005. “Culturable Airborne Fungi in Outdoor Environments in Beijing, China.” Science of the Total Environment 350, no. 1–3: 47–58. 16227072 10.1016/j.scitotenv.2005.01.032

[emi470078-bib-0030] Fatahinia, M. , A. Zarei‐Mahmoudabadi , H. Shokri , and H. Ghaymi . 2018. “Monitoring of Mycoflora in Outdoor Air of Different Localities of Ahvaz, Iran.” Journal of Medical Mycology 28, no. 1: 87–93. 10.1016/j.mycmed.2017.12.002. 29402620

[emi470078-bib-0031] Fuentes‐Gándara, F. , J. Pinedo‐Hernández , E. Gutiérrez , J. Marrugo‐Negrete , and S. Díez . 2021. “Heavy Metal Pollution and Toxicity Assessment in Mallorquin Swamp: A Natural Protected Heritage in the Caribbean Sea, Colombia.” Marine Pollution Bulletin 167: 112271. 33780754 10.1016/j.marpolbul.2021.112271

[emi470078-bib-0032] Fung, F. , and W. G. Hughson . 2003. “Health Effects of Indoor Fungal Bioaerosol Exposure.” Applied Occupational and Environmental Hygiene 18, no. 7: 535–544. 12791550 10.1080/10473220301451

[emi470078-bib-0033] Gallego‐Cartagena, E. , H. Morillas , M. Maguregui , et al. 2020. “A Comprehensive Study of Biofilms Growing on the Built Heritage of a Caribbean Industrial City in Correlation With Construction Materials.” International Biodeterioration & Biodegradation 147: 104874.

[emi470078-bib-0034] Gallego‐Cartagena, E. , H. Morillas , W. Morgado‐Gamero , et al. 2022. “Elemental Imaging Approach to Assess the Ability of Subaerial Biofilms Growing on Constructions Located in Tropical Climates as Potential Biomonitors of Atmospheric Heavy Metals Pollution.” Chemosphere 309: 136743. 36209867 10.1016/j.chemosphere.2022.136743

[emi470078-bib-0035] García‐Bastidas, F. A. , J. C. Quintero‐Vargas , M. Ayala‐Vasquez , et al. 2020. “First Report of *Fusarium* Wilt Tropical Race 4 in Cavendish Bananas Caused by *Fusarium odoratissimum* in Colombia.” Plant Disease 104, no. 3: 994.

[emi470078-bib-0036] Ghajar, F. , P. Holford , E. Cother , and A. Beattie . 2006. “Effects of Ultraviolet Radiation, Simulated or as Natural Sunlight, on Conidium Germination and Appressorium Formation by Fungi With Potential as Mycoherbistats.” Biocontrol Science and Technology 16, no. 5: 451–469. 10.1080/09583150500532642.

[emi470078-bib-0037] Gómez‐Dantés, H. , N. Fullman , H. Lamadrid‐Figueroa , et al. 2016. “Dissonant Health Transition in the States of Mexico, 1990–2013: A Systematic Analysis for the Global Burden of Disease Study 2013.” Lancet 388, no. 10058: 2386–2402. 27720260 10.1016/S0140-6736(16)31773-1

[emi470078-bib-0038] Gong, J. , J. Qi , E. Beibei , Y. Yin , and D. Gao . 2020. “Concentration, Viability and Size Distribution of Bacteria in Atmospheric Bioaerosols Under Different Types of Pollution.” Environmental Pollution 257: 113485. 31708283 10.1016/j.envpol.2019.113485

[emi470078-bib-0040] Gouveia, N. , W. L. Junger , I. Romieu , et al. 2018. “Effects of Air Pollution on Infant and Children Respiratory Mortality in Four Large Latin‐American Cities.” Environmental Pollution 232: 385–391. 28966023 10.1016/j.envpol.2017.08.125

[emi470078-bib-0041] Green, B. J. , E. Yli‐Panula , and E. R. Tovey . 2006. “Halogen Immunoassay, a New Method for the Detection of Sensitization to Fungal Allergens; Comparisons With Conventional Techniques.” Allergology International 55, no. 2: 131–139. 17075249 10.2332/allergolint.55.131

[emi470078-bib-0042] Grewling, Ł. , H. Ribeiro , C. Antunes , et al. 2023. “Outdoor Airborne Allergens: Characterization, Behavior and Monitoring in Europe.” Science of the Total Environment 905: 167042. 10.1016/j.scitotenv.2023.167042. 37709071

[emi470078-bib-0043] Heo, K. J. , S. B. Jeong , C. E. Lim , G. W. Lee , and B. U. Lee . 2023. “Diurnal Variation in Concentration of Culturable Bacterial and Fungal Bioaerosols in Winter to Spring Season.” Atmosphere 14, no. 3: 537.

[emi470078-bib-0044] Hernández‐Flórez, L. J. , G. Aristizabal‐Duque , L. Quiroz , et al. 2013. “Contaminación del aire y enfermedad respiratoria en menores de cinco años de Bogotá, 2007.” Revista de Salud Pública 15, no. 4: 552–565. 25124123

[emi470078-bib-0045] Herrera, C. , and P. Cabrera‐Barona . 2022. “Impact of Perceptions of Air Pollution and Noise on Subjective Well‐Being and Health.” Earth 3, no. 3: 825–838.

[emi470078-bib-0046] Herrera, R. , K. Radon , O. S. von Ehrenstein , S. Cifuentes , D. M. Muñoz , and U. Berger . 2016. “Proximity to Mining Industry and Respiratory Diseases in Children in a Community in Northern Chile: A Cross‐Sectional Study.” Environmental Health 15, no. 1: 1–10. 27266511 10.1186/s12940-016-0149-5 PMC4897925

[emi470078-bib-0047] Huertas, M. E. , R. L. Acevedo‐Barrios , M. Rodríguez , J. Gaviria , R. Arana , and C. Arciniegas . 2018. “Identification and Quantification of Bioaerosols in a Tropical Coastal Region: Cartagena de Indias, Colombia.” Aerosol Science and Engineering 2: 206–215.

[emi470078-bib-0048] IDEAM . 2023. “Atlas Interactivo‐Climatológico.” http://atlas.ideam.gov.co/visorAtlasClimatologico.html.

[emi470078-bib-0049] Jabeen, R. , M. I. Kizhisseri , S. N. Mayanaik , and M. M. Mohamed . 2023. “Bioaerosol Assessment in Indoor and Outdoor Environments: A Case Study From India.” Scientific Reports 13, no. 1: 18066. 37872255 10.1038/s41598-023-44315-z PMC10593752

[emi470078-bib-0050] Jeong, S. B. , H. S. Ko , K. J. Heo , J. H. Shin , and J. H. Jung . 2022. “Size Distribution and Concentration of Indoor Culturable Bacterial and Fungal Bioaerosols.” Atmospheric Environment: X 15: 100182.

[emi470078-bib-0051] Jiménez López, E. , and D. Porras Duran . 2019. “Identificación de los granos de Polen y esporas de hongos presentes en la ciudad de Barranquilla.” Universidad de la Costa. https://repositorio.cuc.edu.co.

[emi470078-bib-0052] Jones, A. M. , and R. M. Harrison . 2004. “The Effects of Meteorological Factors on Atmospheric Bioaerosol Concentrations—A Review.” Science of the Total Environment 326, no. 1–3: 151–180. 15142773 10.1016/j.scitotenv.2003.11.021

[emi470078-bib-0053] Kakde, U. B. 2012. “Fungal Bioaerosols: Global Diversity, Distribution and Its Impact on Human Beings and Agricultural Crops.” Bionano Front 5: 323–329.

[emi470078-bib-0054] Kallawicha, K. , Y.‐J. Tsai , Y.‐C. Chuang , et al. 2015. “The Spatiotemporal Distributions and Determinants of Ambient Fungal Spores in the Greater Taipei Area.” Environmental Pollution 204: 173–180. 10.1016/j.envpol.2015.04.020. 25969377

[emi470078-bib-0055] Kang, S. M. , K. J. Heo , and B. U. Lee . 2015. “Why Does Rain Increase the Concentrations of Environmental Bioaerosols During Monsoon?” Aerosol and Air Quality Research 15, no. 6: 2320–2324.

[emi470078-bib-0056] Kim, K. H. , E. Kabir , and S. A. Jahan . 2018. “Airborne Bioaerosols and Their Impact on Human Health.” Journal of Environmental Sciences 67: 23–35. 10.1016/j.jes.2017.08.027 PMC7128579 29778157

[emi470078-bib-0057] Konishi, N. , M. Kimura , and Y. Takeda . 2024. “Prosociality Predicts Changes in Leisure Activities During the COVID‐19 Pandemic.” Frontiers in Psychology 15: 1320885. 38476389 10.3389/fpsyg.2024.1320885 PMC10927729

[emi470078-bib-0058] Konkel Neabore, L. 2024. “Wake‐Up Call: Rapid Increase in Human Fungal Diseases Under Climate Change.” Environmental Health Perspectives 132, no. 4: 042001. 38648197 10.1289/EHP14722 PMC11034633

[emi470078-bib-0059] Korzeniewska, E. 2011. “Emission of Bacteria and Fungi in the Air From Wastewater Treatment Plants–A Review.” Frontiers in Bioscience‐Scholar 3, no. 2: 393–407. 10.2741/s159 21196384

[emi470078-bib-0060] Kowalski, M. , and J. S. Pastuszka . 2018. “Effect of Ambient Air Temperature and Solar Radiation on Changes in Bacterial and Fungal Aerosols Concentration in the Urban Environment.” Annals of Agricultural and Environmental Medicine 25, no. 2: 259–261. 29936815 10.26444/aaem/75877

[emi470078-bib-0061] Kumari, S. , M. Devi , K. Thakur , B. Minhas , A. K. Bhatt , and N. Kaushik . 2024. “Impact of Anthropogenic Activities on Microbial Diversity and Soil Health.” In Advancements in Microbial Biotechnology for Soil Health. Microorganisms for Sustainability, edited by R. K. Bhatia and A. Walia , vol. 50. Springer. 10.1007/978-981-99-9482-3_11.

[emi470078-bib-0062] Laumbach, R. J. , and H. M. Kipen . 2005. “Bioaerosols and Sick Building Syndrome: Particles, Inflammation, and Allergy.” Current Opinion in Allergy and Clinical Immunology 5, no. 2: 135–139. 15764903 10.1097/01.all.0000162305.05105.d0

[emi470078-bib-0063] Le, T. C. , and C. J. Tsai . 2021. “Inertial Impaction Technique for the Classification of Particulate Matters and Nanoparticles: A Review.” Kona: Powder Science and Technology in Japan 38: 42–63.

[emi470078-bib-0064] Lee, B. U. , S. H. Yun , J. H. Jung , and G. N. Bae . 2010. “Effect of Relative Humidity and Variation of Particle Number Size Distribution on the Inactivation Effectiveness of Airborne Silver Nanoparticles Against bacteria Bioaerosols Deposited on a Filter.” Journal of Aerosol Science 41, no. 5: 447–456.

[emi470078-bib-0065] Lee, G. , and K. Yoo . 2022. “A Review of the Emergence of Antibiotic Resistance in Bioaerosols and Its Monitoring Methods.” Reviews in Environmental Science and Bio/Technology 21, no. 3: 799–827. 10.1007/s11157-022-09622-3 PMC9169023 35694630

[emi470078-bib-0066] Lee, J. H. , and W. K. Jo . 2006. “Characteristics of Indoor and Outdoor Bioaerosols at Korean High‐Rise Apartment Buildings.” Environmental Research 101, no. 1: 11–17. 16199028 10.1016/j.envres.2005.08.009

[emi470078-bib-0067] Li, B. , W. Tan , L. Wen , et al. 2020. “Anthropogenic Habitat Alternation Significantly Decreases α‐and β‐Diversity of Benthopelagic Metacommunity in a Large Floodplain Lake.” Hydrobiologia 847: 293–307.

[emi470078-bib-0068] Li, H. , H. Pan , Y. Lei , H. Wang , S. Li , and C. Xiao . 2024. “Spinal Infection Caused by *Aspergillus flavus* in a Diabetic: A Case Report and Literature Review.” Frontiers in Medicine 11: 1348203. 38371517 10.3389/fmed.2024.1348203 PMC10869514

[emi470078-bib-0069] Li, L. , J. Ma , K. Yang , F. Chai , J. Liu , and X. Guo . 2021. “Microbial Aerosol Particles in Four Seasons of Sanitary Landfill Site: Molecular Approaches, Traceability and Risk Assessment.” Journal of Environmental Sciences 108: 120–133. 10.1016/j.jes.2021.01.013 34465426

[emi470078-bib-0070] Li, Y. , W. Wang , X. Guo , et al. 2015. “Assessment of Airborne Bacteria and Fungi in Various University Indoor Environments: A Case Study in Chang'an University, China.” Environmental Engineering Science 32, no. 4: 273–283.

[emi470078-bib-0071] Liu, Z. , P. Zhang , Y. Li , et al. 2021. “Assessment of Spatial Concentration Variation and Deposition of Bioaerosol in a Dental Clinic During Oral Cleaning.” Building and Environment 202: 108024.

[emi470078-bib-0072] Madhwal, S. , V. Prabhu , S. Sundriyal , and V. Shridhar . 2020a. “Distribution, Characterization and Health Risk Assessment of Size Fractionated Bioaerosols at an Open Landfill Site in Dehradun, India.” Atmospheric Pollution Research 11, no. 1: 156–169.

[emi470078-bib-0073] Madhwal, S. , V. Prabhu , S. Sundriyal , and V. Shridhar . 2020b. “Ambient Bioaerosol Distribution and Associated Health Risks at a High Traffic Density Junction at Dehradun City, India.” Environmental Monitoring and Assessment 192: 1–15. 10.1007/s10661-020-8158-9 PMC7087893 32086610

[emi470078-bib-0074] Madsen, A. M. , M. Raulf , P. Duquenne , et al. 2021. “Review of Biological Risks Associated With the Collection of Municipal Wastes.” Science of the Total Environment 791: 148287. 34139489 10.1016/j.scitotenv.2021.148287

[emi470078-bib-0075] Maji, K. J. , and A. Namdeo . 2021. “Continuous Increases of Surface Ozone and Associated Premature Mortality Growth in China During 2015–2019.” Environmental Pollution 269: 116183. 33288298 10.1016/j.envpol.2020.116183

[emi470078-bib-0076] Mentese, S. , M. Arisoy , A. Y. Rad , and G. Güllü . 2009. “Bacteria and Fungi Levels in Various Indoor and Outdoor Environments in Ankara, Turkey.” Chemosphere 74, no. 3: 377–381.18996567

[emi470078-bib-0077] Metelmann, S. , K. Pattni , L. Brierley , et al. 2021. “Impact of Climatic, Demographic and Disease Control Factors on the Transmission Dynamics of COVID‐19 in Large Cities Worldwide.” One Health 12: 100221. 33558848 10.1016/j.onehlt.2021.100221 PMC7857042

[emi470078-bib-0078] Mo, L. , A. Zanella , A. Squartini , et al. 2024. “Anthropogenic vs. Natural Habitats: Higher Microbial Biodiversity Pays the Trade‐Off of Lower Connectivity.” Microbiological Research 282: 127651. 38430888 10.1016/j.micres.2024.127651

[emi470078-bib-0079] Mohammad, N. , A. Huguenin , A. Lefebvre , et al. 2024. “Nosocomial Transmission of *Aspergillus flavus* in a Neonatal Intensive Care Unit: Long‐Term Persistence in Environment and Interest of MALDI–ToF Mass‐Spectrometry Coupled With Convolutional Neural Network for Rapid Clone Recognition.” Medical Mycology 62, no. 1: myad136. 38142226 10.1093/mmy/myad136

[emi470078-bib-0080] Morgado Gamero, W. B. , D. Agudelo‐Castañeda , M. C. Ramírez , et al. 2018. “Hospital Admission and Risk Assessment Associated to Exposure of Fungal Bioaerosols at a Municipal Landfill Using Statistical Models.” In Intelligent Data Engineering and Automated Learning–IDEAL 2018: 19th International Conference, Madrid, Spain, November 21–23, 2018, Proceedings, Part II 19, edited by H. Yin , D. Camacho , P. Novais , and A. Tallón‐Ballesteros , 210–218. Springer International Publishing.

[emi470078-bib-0081] Morgado Gamero, W. B. , M. C. Ramírez , A. Parody , A. Viloria , M. H. A. López , and S. J. Kamatkar . 2018. “Concentrations and Size Distributions of Fungal Bioaerosols in a Municipal Landfill.” In Data min. Big Data: Third International Conference, DMBD 2018, Shanghai, China, June 17–22, 2018, Proceedings 3, 244–253. Springer International Publishing.

[emi470078-bib-0082] Morgado‐Gamero, W. B. , L. Hernández , J. Medina , et al. 2025. “Antibiotic‐Resistant Bacteria Aerosol in a Caribbean Coastal City: Pre‐and Post‐COVID‐19 Lockdown.” Science of the Total Environment 959: 178158. 39721525 10.1016/j.scitotenv.2024.178158

[emi470078-bib-0083] Morgado‐Gamero, W. B. , M. Mendoza Hernández , M. Castillo Ramírez , et al. 2019. “Antibiotic Resistance of Airborne Viable Bacteria and Size Distribution in Neonatal Intensive Care Units.” International Journal of Environmental Research and Public Health 16, no. 18: 3340. 31510047 10.3390/ijerph16183340 PMC6765827

[emi470078-bib-0084] Morgado‐Gamero, W. B. , A. Parody , J. Medina , L. A. Rodríguez‐Villamizar , and D. Agudelo‐Castañeda . 2021. “Multi‐Antibiotic Resistant Bacteria in Landfill Bioaerosols: Environmental Conditions and Biological Risk Assessment.” Environmental Pollution 290: 118037. 34482243 10.1016/j.envpol.2021.118037

[emi470078-bib-0085] Mosalaei, S. , H. Amiri , A. Rafiee , A. Abbasi , A. N. Baghani , and M. Hoseini . 2021. “Assessment of Fungal Bioaerosols and Particulate Matter Characteristics in Indoor and Outdoor Air of Veterinary Clinics.” Journal of Environmental Health Science and Engineering 19, no. 2: 1773–1780. 34900306 10.1007/s40201-021-00732-8 PMC8617105

[emi470078-bib-0086] Nguyen, X. D. , Y. Zhao , J. D. Evans , et al. 2021. “Evaluation of Bioaerosol Samplers for Collecting Airborne *E. coli* Carried by Dust Particles From Poultry Litter.” In 2021 ASABE Annual International Virtual Meeting, 1. American society of agricultural and biological engineers.

[emi470078-bib-0087] Niazi, S. , M. S. Hassanvand , A. H. Mahvi , et al. 2015. “Assessment of Bioaerosol Contamination (Bacteria and Fungi) in the Largest Urban Wastewater Treatment Plant in the Middle East.” Environmental Science and Pollution Research 22: 16014–16021. 26062460 10.1007/s11356-015-4793-z

[emi470078-bib-0088] Nicolaisen, M. , J. S. West , R. Sapkota , G. G. Canning , C. Schoen , and A. F. Justesen . 2017. “Fungal Communities Including Plant Pathogens in Near Surface Air Are Similar Across Northwestern Europe.” Frontiers in Microbiology 8: 1729. 28943873 10.3389/fmicb.2017.01729 PMC5596660

[emi470078-bib-0089] Nolte, C. 2016. “Identifying Challenges to Enforcement in Protected Areas: Empirical Insights From 15 Colombian Parks.” Oryx 50, no. 2: 317–322.

[emi470078-bib-0090] Norouzian Baghani, A. , A. Sorooshian , M. Delikhoon , R. Nabizadeh , S. Nazmara , and R. Bakhtiari . 2021. “Pollution Characteristics and Noncarcinogenic Risk Assessment of Fungal Bioaerosol in Different Processing Units of Waste Paper and Cardboard Recycling Factory.” Toxin Reviews 40, no. 4: 752–763.

[emi470078-bib-0091] Olivares, B. O. , J. C. Rey , D. Lobo , J. A. Navas‐Cortés , J. A. Gómez , and B. B. Landa . 2021. “ *Fusarium* Wilt of Bananas: A Review of Agro‐Environmental Factors in the Venezuelan Production System Affecting Its Development.” Agronomy 11, no. 5: 986.

[emi470078-bib-0092] Ortega Rosas, C. I. , M. D. C. Calderón‐Ezquerro , and O. G. Gutiérrez‐Ruacho . 2020. “Fungal Spores and Pollen Are Correlated With Meteorological Variables: Effects in Human Health at Hermosillo, Sonora, Mexico.” International Journal of Environmental Health Research 30, no. 6: 677–695. 31161773 10.1080/09603123.2019.1625031

[emi470078-bib-0093] Pastuszka, J. S. , E. Marchwinska‐Wyrwal , and A. Wlazlo . 2005. “Bacterial Aerosol in Silesian Hospitals: Preliminary Results.” Polish Journal of Environmental Studies 14, no. 6: 883.

[emi470078-bib-0094] Patel, T. Y. , M. Buttner , D. Rivas , C. Cross , D. A. Bazylinski , and J. Seggev . 2018. “Variation in Airborne Fungal Spore Concentrations Among Five Monitoring Locations in a Desert Urban Environment.” Environmental Monitoring and Assessment 190: 1–10. 10.1007/s10661-018-7008-5 PMC6208991 30338422

[emi470078-bib-0095] Penack, O. , G. Tridello , U. Salmenniemi , et al. 2024. “Influence of Invasive Aspergillosis During Acute Leukaemia Treatment on Survival After Allogeneic Stem Cell Transplantation: A Prospective Study of the EBMT Infectious Diseases Working Party.” EClinicalMedicine 67: 102393. 10.1016/j.eclinm.2023.102393. 38152413 PMC10751840

[emi470078-bib-0096] Portz, L. , R. P. Manzolli , C. F. F. de Andrade , D. A. Villate Daza , and J. Alcántara‐Carrió . 2020. “Assessment of Heavy Metals Pollution (Hg, Cr, cd, Ni) in the Sediments of Mallorquin Lagoon‐Barranquilla, Colombia.” Journal of Coastal Research 95, no. SI: 158–162.

[emi470078-bib-0097] Priyamvada, H. , R. K. Singh , M. Akila , R. Ravikrishna , R. S. Verma , and S. S. Gunthe . 2017. “Seasonal Variation of the Dominant Allergenic Fungal Aerosols—One Year Study From Southern Indian Region.” Scientific Reports 7, no. 1: 11171. 28894264 10.1038/s41598-017-11727-7 PMC5593913

[emi470078-bib-0098] Qi, Y. , Y. Li , W. Xie , et al. 2020. “Temporal‐Spatial Variations of Fungal Composition in PM_2.5_ and Source Tracking of Airborne Fungi in Mountainous and Urban Regions.” Science of the Total Environment 708: 135027. 31787277 10.1016/j.scitotenv.2019.135027

[emi470078-bib-0099] Raposo Puglia, D. , J. Á. Raposo Puglia , E. García‐Cabrera , F. Morales , J. C. Camacho‐Vega , and Á. Vilches‐Arenas . 2024. “Risk Factors and Environmental Preventive Actions for Aspergillosis in Patients With Hematological Malignancies.” Clinics and Practice 14, no. 1: 280–292. 38391408 10.3390/clinpract14010022 PMC10888107

[emi470078-bib-0100] Robichaud, A. 2020. “An Overview of Selected Emerging Outdoor Airborne Pollutants and Air Quality Issues: The Need to Reduce Uncertainty About Environmental and Human Impacts.” Journal of the Air & Waste Management Association 70, no. 4: 341–378. 31994992 10.1080/10962247.2020.1723738

[emi470078-bib-0101] Rodríguez‐Camargo, L. A. , R. J. Sierra‐Parada , and L. C. Blanco‐Becerra . 2020. “Análisis espacial de las concentraciones de PM_2.5_ en Bogotá según los valores de las guías de la calidad del aire de la Organización Mundial de la Salud para enfermedades cardiopulmonares, 2014‐2015.” Biomédica 40, no. 1: 137–152. 32220170 10.7705/biomedica.4719 PMC7357390

[emi470078-bib-0102] Rodríguez‐Gómez, C. , C. Ramírez‐Romero , F. Córdoba , et al. 2020. “Characterization of Culturable Airborne Microorganisms in the Yucatan Península.” Atmospheric Environment 223: 117183.

[emi470078-bib-0103] Rodríguez‐Villamizar, L. A. , L. C. Belalcázar‐Cerón , M. P. Castillo , and D. M. Agudelo‐Castañeda . 2022. “Avoidable Mortality Due to Long‐Term Exposure to PM_2.5_ in Colombia 2014–2019.” Environmental Health 21, no. 1: 137. 36564760 10.1186/s12940-022-00947-8 PMC9789551

[emi470078-bib-0104] Rodríguez‐Villamizar, L. A. , L. C. Belalcazar‐Ceron , J. A. Fernández‐Nino , et al. 2021. “Air Pollution, Sociodemographic and Health Conditions Effects on COVID‐19 Mortality in Colombia: An Ecological Study.” Science of the Total Environment 756: 144020. 33279185 10.1016/j.scitotenv.2020.144020 PMC7688425

[emi470078-bib-0105] Rotem, J. , and H. J. Aust . 1991. “The Effect of Ultraviolet and Solar Radiation and Temperature on Survival of Fungal Propagules.” Journal of Phytopathology 133, no. 1: 76–84. 10.1111/j.1439-0434.1991.tb00139.x.

[emi470078-bib-0106] Sajjad, B. , K. Rasool , A. Siddique , et al. 2023. “Size‐Resolved Ambient Bioaerosols Concentration, Antibiotic Resistance, and Community Composition During Autumn and Winter Seasons in Qatar.” Environmental Pollution 336: 122401. 37598930 10.1016/j.envpol.2023.122401

[emi470078-bib-0107] Šantl‐Temkiv, T. , B. Sikoparija , T. Maki , et al. 2020. “Bioaerosol Field Measurements: Challenges and Perspectives in Outdoor Studies.” Aerosol Science and Technology 54, no. 5: 520–546.

[emi470078-bib-0108] Schöttl, S. E. , M. Schnitzer , L. Savoia , and M. Kopp . 2022. “Physical Activity Behavior During and After COVID‐19 Stay‐At‐Home Orders—A Longitudinal Study in the Austrian, German, and Italian Alps.” Frontiers in Public Health 10: 901763. 35712287 10.3389/fpubh.2022.901763 PMC9194442

[emi470078-bib-0109] Schwab, C. J. , and D. C. Straus . 2004. “The Roles of *Penicillium* and *Aspergillus* in Sick Building Syndrome.” Advances in Applied Microbiology 55: 215–240. 15350796 10.1016/S0065-2164(04)55008-6

[emi470078-bib-0110] Seidel, D. , S. Wurster , J. D. Jenks , et al. 2024. “Impact of Climate Change and Natural Disasters on Fungal Infections.” Lancet Microbe 5, no. 6: 594–605. 10.1016/S2666-5247(24)00039-9 38518791

[emi470078-bib-0111] Seinfeld, J. H. , and S. N. Pandis . 2016. Atmospheric Chemistry and Physics: From Air Pollution to Climate Change. John Wiley & Sons.

[emi470078-bib-0112] Singh, M. , and A. Hays . 2016. “Indoor and Outdoor Allergies.” Primary Care 43, no. 3: 451–463. 10.1016/j.pop.2016.04.013. 27545734

[emi470078-bib-0113] Stetzenbach, L. D. , M. P. Buttner , and P. Cruz . 2004. “Detection and Enumeration of Airborne Biocontaminants.” Current Opinion in Biotechnology 15, no. 3: 170–174. 15193322 10.1016/j.copbio.2004.04.009

[emi470078-bib-0114] Sykes, J. E. , and S. Rankin . 2014. “Chapter 4—Isolation and Identification of Fungi.” In Canine and Feline Infectious Diseases, edited by J. E. Sykes , 29–36. Elsevier Inc.

[emi470078-bib-0115] van Rhijn, N. , I. S. Storer , M. Birch , J. D. Oliver , M. J. Bottery , and M. J. Bromley . 2024. “ *Aspergillus fumigatus* Strains That Evolve Resistance to the Agrochemical Fungicide Ipflufenoquin In Vitro Are Also Resistant to Olorofim.” Nature Microbiology 9, no. 1: 29–34. 10.1038/s41564-023-01542-4 PMC10769868 38151646

[emi470078-bib-0116] Verweij, P. E. , M. C. Arendrup , A. Alastruey‐Izquierdo , et al. 2022. “Dual Use of Antifungals in Medicine and Agriculture: How Do We Help Prevent Resistance Developing in Human Pathogens?” Drug Resistance Updates 65: 100885. 36283187 10.1016/j.drup.2022.100885 PMC10693676

[emi470078-bib-0117] Walti, L. , C. Crone , R. Bitterman , et al. 2024. “Early and Late Invasive Aspergillosis in the First Year Post Lung Transplant (LT)‐A Multicentre Study.” Journal of Heart and Lung Transplantation 43, no. 4: S71.

[emi470078-bib-0118] Wan, L. , X. Cai , M. Ling , J. Kan , M. Yin , and H. Wang . 2024. “Evaluation of the JF5‐Based *Aspergillus* Galactomannoprotein Lateral Flow Device for Diagnosing Invasive Aspergillosis in Cancer Patients.” European Journal of Clinical Microbiology & Infectious Diseases 43: 1–9. 38625450 10.1007/s10096-024-04830-x

[emi470078-bib-0120] Wei, D. L. , J. H. Chen , S. C. Jong , and H. D. Shen . 1993. “Indoor Airborne *Penicillium* Species in Taiwan.” Current Microbiology 26: 137–140.

[emi470078-bib-0121] Whittaker, G. , M. Taylor , M. Chamula , F. Granato , H. Balata , and C. Kosmidis . 2024. “Chronic Pulmonary Aspergillosis After Surgical Treatment for Non‐Small Cell Lung Cancer—An Analysis of Risk Factors and Clinical Outcomes.” Journal of Fungi 10, no. 5: 335. 38786690 10.3390/jof10050335 PMC11121761

[emi470078-bib-0122] Zhang, T. , G. Engling , C. Y. Chan , et al. 2010. “Contribution of Fungal Spores to Particulate Matter in a Tropical Rainforest.” Environmental Research Letters 5, no. 2: 24010.

[emi470078-bib-0123] Zheng, F. , J. Gao , M. Tang , et al. 2024. “Urbanization Reduces the Stability of Soil Microbial Community by Reshaping the Diversity and Network Complexity.” Chemosphere 364: 143177. 39182733 10.1016/j.chemosphere.2024.143177

[emi470078-bib-0124] Zhong, X. , J. Qi , H. Li , L. Dong , and D. Gao . 2016. “Seasonal Distribution of Microbial Activity in Bioaerosols in the Outdoor Environment of the Qingdao Coastal Region.” Atmospheric Environment 140: 506–513.

[emi470078-bib-0125] Zuriegat, Q. , Y. Zheng , H. Liu , Z. Wang , and Y. Yun . 2021. “Current Progress on Pathogenicity‐Related Transcription Factors in *Fusarium oxysporum* .” Molecular Plant Pathology 22, no. 7: 882–895. 33969616 10.1111/mpp.13068 PMC8232035

