# Supplementary material for: Urbanisation and Lockdown Impact on Airborne Fungal Communities in Tropical Landscapes: A Comparative Study of Urban and Peri‐Urban Environments
Source: Environ Microbiol Rep. 2025 May 13;17(3):e70078. doi: 10.1111/1758-2229.70078 (PMC12074671; doi:10.1111/1758-2229.70078)
Supplement: Supplementary file 1 — Data S1. Supporting Information. [file EMI4-17-e70078-s001.docx]

**Urbanization and lockdown impact on airborne fungal communities in tropical landscapes: a comparative study of urban and peri-urban environments**

Euler Gallego-Cartagena^1^*, Wendy Morgado-Gamero^2,3^, Iuleder de Moya-Hernández^4^, Carlos Díaz-Uribe^4^, Alexander Parody^5^, Héctor Morillas^6^, Brayan Bayona-Pacheco^7^, Gabrielle Pellegrin^8^, Dayana Agudelo-Castañeda^9^

^1^ Department of Civil and Environmental, Universidad de la Costa, Calle 58 #55-66, 080002, Barranquilla, Colombia

^2^ Department of Exact and Natural Sciences, Universidad de la Costa, Colombia

^3^Department of Biology, McGill University, Montreal, Quebec, Canada

^4^Chemistry Program, Faculty of Basic Sciences, Universidad del Atlántico, Puerto Colombia, Atlántico, Colombia

^5^Faculty of Engineering, Universidad Libre, Colombia

#### ^6^ Department of Didactic of Mathematics, Experimental and Social Sciences, Faculty of Education and Sport, University of the Basque Country, Basque Country, Spain.

^7^Department of Medicine, Division of Health Sciences, Universidad del Norte, Barranquilla, Colombia

^8^IRD Institut de recherche pour le développement, Marseille, France

^9^Department of Civil and Environmental Engineering, Universidad del Norte, Puerto Colombia, AMB Barranquilla, Colombia

**SUPPLEMENTARY MATERIAL**


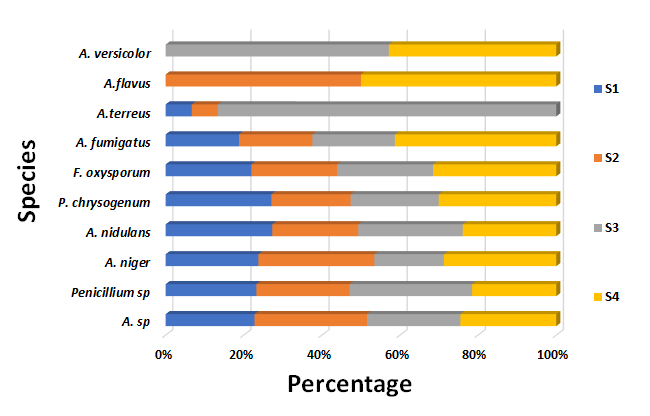


**Fig. S1. Percentage distribution of concentrations of fungal bioaerosols found and identified by monitoring station**


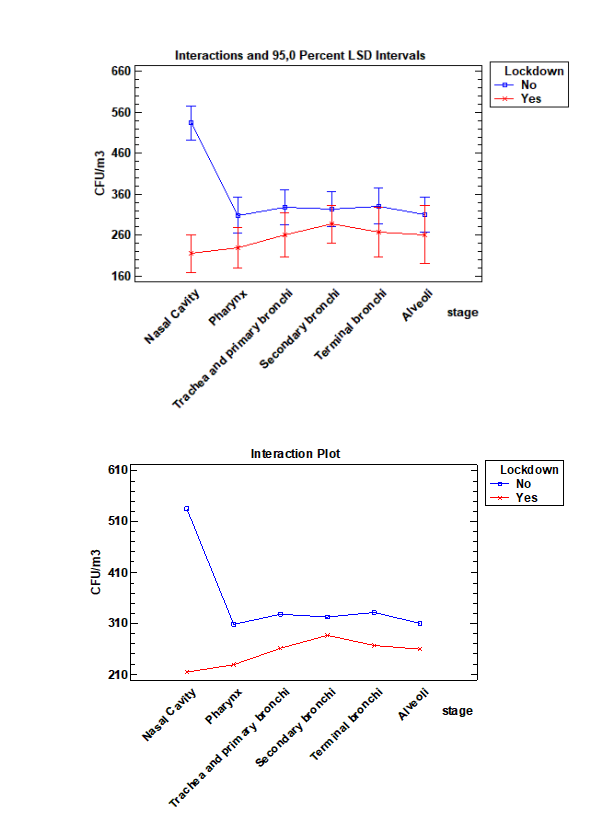


**Fig. S2.** Interaction plot using Fisher's LSD test. It can be seen that the concentration of fungi in the nasal cavity was much higher before isolation compared to after isolation, in the rest of the stages there are no statistically significant differences (LSD test intervals overlap).

**Table S1.** Analysis of Variance for CFU/m^3^

|  | **Squares Sum** | **Df** | **Mean Square** | **F-Ratio** | **P-Value** |
| --- | --- | --- | --- | --- | --- |
| **Model** | 1.66x10^6^ | 29 | 571324, | 5,81 | 0,0000 |
| **Residual** | 1.05x10^6^ | 1070 | 98280,6 |  |  |
| **Total (Corrected)** | 1.22 x10^8^ | 1099 |  |  |  |

**Table S2.** Bayesian Neural Network Classifier - Concentration (Selection)

**Classification Factor:** Concentration

**Factors:** Monitoring, Session, Station or Point, Temperature, Humidity, Wind Speed (M/S), Wind Direction, Stage

**Prior Probabilities:** Proportional to occurrence in the training set

**Error Costs:** Equal for all cases

**Variable Selection:** Selection

**Number of cases in the training set:** 626

**Number of cases in the validation set:** 173

**Spacing Parameter Used:** 0.298438 (optimized through jackknifing during training)

**Table S3. Training set**

|  |  | **Percent correctly** |
| --- | --- | --- |
| **Concentration** | **Member** | **Classified** |
| High | 295 | 58,9831 |
| Low | 331 | 95,7704 |
| Total | 626 | 78,4345 |

**Table S4. Validation set**

|  |  | **Percent correctly** |
| --- | --- | --- |
| **Concentration** | **Member** | **Classified** |
| High | 98 | 63,2653 |
| Low | 75 | 94,6667 |
| Total | 173 | 76,8786 |

**Table S5 Analysis of Variance for CFU/m3 - Type III Sums of Squares**

| ***Source*** | ***Sum of Squares*** | ***Df*** | ***Mean Square*** | ***F-Ratio*** | ***P-Value*** |
| --- | --- | --- | --- | --- | --- |
| **MAIN EFFECTS** |  |  |  |  |  |
| **A:Lockdown** | **2,61346E6** | **1** | **2,61346E6** | **24,96** | **0,0000** |
| **B:stage** | **1,31416E6** | **5** | **262831,** | **2,51** | **0,0286** |
| **INTERACTIONS** |  |  |  |  |  |
| **AB** | **2,87681E6** | **5** | **575362,** | **5,49** | **0,0001** |
| **RESIDUAL** | **1,13921E8** | **1088** | **104707,** |  |  |
| **TOTAL (CORRECTED)** | **1,21729E8** | **1099** |  |  |  |
